# JAK2 is dispensable for maintenance of JAK2 mutant B-cell acute lymphoblastic leukemias

**DOI:** 10.1101/gad.307504.117

**Published:** 2018-06-01

**Authors:** Sang-Kyu Kim, Deborah A. Knight, Lisa R. Jones, Stephin Vervoort, Ashley P. Ng, John F. Seymour, James E. Bradner, Michaela Waibel, Lev Kats, Ricky W. Johnstone

**Affiliations:** 1 The Peter MacCallum Cancer Centre, Melbourne, 3000 Victoria, Australia;; 2 The Sir Peter MacCallum Department of Oncology, University of Melbourne, Parkville, 3052 Victoria, Australia;; 3 Division of Cancer and Haematology, The Walter and Eliza Hall Institute of Medical Research, Parkville, 3052 Victoria, Australia;; 4 Department of Medical Biology, University of Melbourne, Parkville, 3010 Victoria, Australia;; 5 Novartis Institutes for BioMedical (NIBR) Research, Cambridge, Massachusetts 02139, USA

**Keywords:** JAK2, B-cell acute lymphoblastic leukemia, ruxolitinib, oncogene addiction, c-Myc, JQ1, BET bromodomain inhibitor

## Abstract

Activating JAK2 point mutations are implicated in the pathogenesis of myeloid and lymphoid malignancies, including high-risk B-cell acute lymphoblastic leukemia (B-ALL). In preclinical studies, treatment of JAK2 mutant leukemias with type I JAK2 inhibitors (e.g., Food and Drug Administration [FDA]-approved ruxolitinib) provided limited single-agent responses, possibly due to paradoxical JAK2^Y1007/1008^ hyperphosphorylation induced by these agents. To determine the importance of mutant JAK2 in B-ALL initiation and maintenance, we developed unique genetically engineered mouse models of B-ALL driven by overexpressed Crlf2 and mutant Jak2, recapitulating the genetic aberrations found in human B-ALL. While expression of mutant Jak2 was necessary for leukemia induction, neither its continued expression nor enzymatic activity was required to maintain leukemia survival and rapid proliferation. CRLF2/JAK2 mutant B-ALLs with sustained depletion or pharmacological inhibition of JAK2 exhibited enhanced expression of c-Myc and prominent up-regulation of c-Myc target genes. Combined indirect targeting of c-Myc using the BET bromodomain inhibitor JQ1 and direct targeting of JAK2 with ruxolitinib potently killed JAK2 mutant B-ALLs.

## Abstract (precis)

Kim et al. show that while expression of mutant Jak2 is necessary for B-cell acute lymphoblastic leukemia induction, neither its continued expression nor enzymatic activity is required to maintain leukemia survival and rapid proliferation.

Relapsed B-cell acute lymphoblastic leukemia (B-ALL) is one of the leading causes of cancer-related mortality in children and young adults (Hunger and Mullighan 2015). Between 15% and 30% of all childhood B-ALLs, termed “Ph-like” B-ALL, exhibit a gene expression profile similar to that of *BCR–ABL1*-expressing B-ALL and are associated with poor prognosis (Den Boer et al. 2009; Mullighan et al. 2009b; Roberts et al. 2014). Little is currently known about the alternative treatment options in the up-front setting or recurrent disease for this cohort that is at high-risk of relapse, highlighting an urgent need for novel therapeutic strategies in this subset of B-ALL (Cario et al. 2010; Ensor et al. 2011).

Recent genomic studies revealed that Ph-like B-ALLs comprise a spectrum of somatic variations in genes encoding protein kinases and cytokine receptor signaling molecules (Roberts et al. 2012, 2014). The majority of cases in Ph-like B-ALL harbors *CRLF2* gene rearrangements (either *IGH–CRLF2* or *P2RY8–CRLF2*) leading to cell surface overexpression of CRLF2, a component of the heterodimeric cytokine receptor complex for thymic stromal lymphopoietin (TSLP) (Mullighan et al. 2009a; Russell et al. 2009). Approximately 50% of *CRLF2*-rearranged B-ALLs have concomitant activating point mutations at highly conserved regions of *JAK2* (exon 16), the most common of which is the B-ALL-exclusive activating *JAK2*^R683G^ alteration (Mullighan et al. 2009a; Harvey et al. 2010; Hertzberg et al. 2010).

Somatic *JAK2* point mutations have been shown previously to be bona fide cancer-initiating lesions in myeloproliferative neoplasms (MPNs) (Baxter et al. 2005; Levine et al. 2005; Lacout et al. 2006; Wernig et al. 2006), where ∼95% of polycythemia vera (PV) and 50% of essential thrombocythemia (ET)/primary myelofibrosis (PMF) cases harbor the recurrent activating *JAK2*^V617F^ alteration (Baxter et al. 2005; James et al. 2005; Kralovics et al. 2005; Levine et al. 2005). This discovery provided a rationale for targeting deregulated JAK2 signaling as a treatment strategy in MPNs, prompting the rapid development and clinical application of type I (ATP-competitive) JAK2 kinase inhibitors (JAK2is), including Food and Drug Administration (FDA)-approved ruxolitinib (Verstovsek et al. 2012). Despite the lack of molecular response observed in the majority of ruxolitinib-treated MPN patients (Harrison et al. 2012; Verstovsek et al. 2012), genetic studies have demonstrated that MPN cells are indeed reliant on aberrant JAK/STAT signaling, underscoring its relevance as a therapeutic target in this disease (Bhagwat et al. 2014).

Mutant JAK2-expressing B-ALLs also exhibit deregulated JAK/STAT signaling (Tasian et al. 2012), suggesting that inhibition of this hyperactive signaling network may be therapeutically beneficial. However, B-ALLs exhibit limited inherent sensitivity to structurally distinct type I JAK2is (including ruxolitinib) (Maude et al. 2012; Tasian et al. 2012), which induce paradoxical JAK2^Y1007/1008^ activation loop hyperphosphorylation despite potent suppression of key JAK2 substrates, including STAT5 (Weigert et al. 2012; Wu et al. 2015). The putative maintenance of JAK2 signaling following type I JAK2i treatment of B-ALLs led to the development of alternative strategies to antagonize JAK2 kinase activity (Weigert et al. 2012; Wu et al. 2015). Accordingly, the type II (non-ATP-competitive) JAK2i CHZ868 that potently abrogates pJAK2^Y1007/1008^ and downstream pSTAT5^Y694^ was shown recently to exhibit efficacy superior to that of type I JAK2is in inducing apoptosis of *CRLF2*-rearranged/JAK2 mutant B-ALLs (Wu et al. 2015).

Here, we developed genetically engineered mouse models (GEMMs) of CRLF2/JAK2 mutant B-ALLs and used a pharmacogenomics approach to delineate the functional role of JAK2 in these leukemias and human *CRLF2*-rearranged/JAK2^I682F^-expressing MHH-CALL4 B-ALLs. We demonstrate an essential role for mutant Jak2 in cooperating with overexpressed surface Crlf2 to initiate B-ALL development. However, pharmacological JAK2 inhibition using type I JAK2i ruxolitinib or genetic depletion or deletion of JAK2 resulted in only a marginal decrease in cell proliferation with little or no induction of cell death. Treatment of these leukemias with CHZ868 resulted in apoptosis (Wu et al. 2015); however, we demonstrate that this compound remained potent even in JAK2-depleted CRLF2/JAK2 mutant B-ALLs, indicating a putative off-target effector mechanism. Consequent interrogation for mechanisms underlying the maintenance of proliferation of CRLF2/mutant JAK2-driven B-ALLs following sustained genetic or pharmacological targeting of JAK2 revealed enhanced c-Myc protein expression and a compensatory c-Myc-driven gene expression signature that may have mediated prolonged growth of these cells. Target depletion of c-Myc in JAK2 mutant B-ALLs using the BET inhibitor (JQ1) or RNAi combined with direct inhibition of JAK2 using ruxolitinib potently abrogated c-Myc expression and synergistically killed CRLF2/JAK2 mutant B-ALLs. Cotreatment of mice bearing CRLF2/JAK2 mutant B-ALLs with JQ1 and ruxolitinib further prolonged overall survival of mice compared with single-agent treatment.

## Results

### Pharmacological inhibition or genetic depletion of JAK2 in MHH-CALL4 B-ALL cells induces an anti-proliferative response and not apoptosis

**Figure 1.**
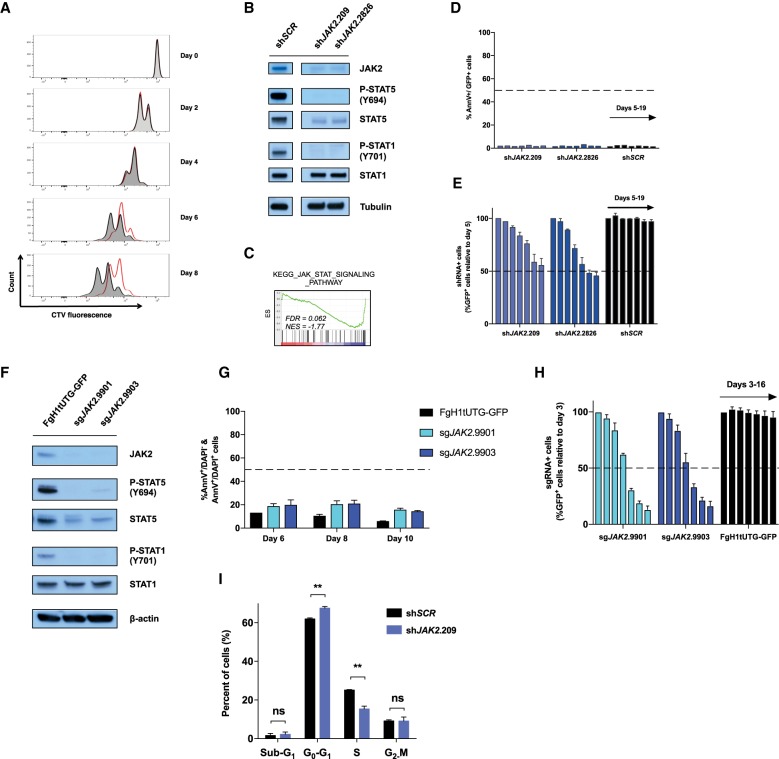
Pharmacological inhibition or genetic depletion of JAK2 in MHH-CALL4 B-ALL cells induces an anti-proliferative response and not apoptosis. (*A*) CellTrace violet (CTV)-stained MHH-CALL4 cells were treated with vehicle (dimethylsulfoxide [DMSO]; black) or 1000 nM ruxolitinib (red). Cells were serially passaged for up to 8 d and subjected to assessment of their proliferative capacity by flow cytometry every 2 d. Drugs were replenished to the indicated concentration every 2 d. Data are representative of *n* = 3 performed in duplicate. (*B*) MHH-CALL4 cells were transduced with constitutive (pLMS-GFP) retroviral vectors harboring shRNAs targeting *JAK2* (sh*JAK2*.209 or sh*JAK2*.2826) or scrambled (sh*SCR*). Immunoblotting was performed against the indicated targets using lysates from shRNA^+^ cells (GFP^+^) at day 5 after transduction. Tubulin served as loading control. (*C*) Gene set enrichment analysis (GSEA) from 3′RNA sequencing (3′RNA-seq) analysis demonstrates negative enrichment of the indicated JAK/STAT gene set in MHH-CALL4-pLMS-sh*JAK2*.209 cells relative to MHH-CALL4-pLMS-sh*SCR* cells at day 5 after transduction. (NES) Normalized enrichment score; (FDR) false discovery rate. (*D*,*E*) Transduced and nontransduced populations from *B* were passaged in vitro for 19 d. (*D*) The percentage of Annexin V^+^/GFP^+^ cells was assessed by flow cytometry (individual bars represent days 5, 8, 9, 12, 14, 16, and 19 after transduction). (*E*) The percentage of shRNA^+^ cells (GFP^+^) was assessed by flow cytometry, and data were normalized to day 5. (*F*) Immunoblotting against the indicated target was performed against MHH–CALL4–Cas9 cells transduced with lentiviral vector (FgH1tUTG-GFP) expressing single guide RNAs (sgRNAs) targeting *JAK2* (sh*JAK2*.9901 or sh*JAK2*.9903) at day 6 after sgRNA induction. β-Actin served as loading control. (*G*) Transduced populations from *F* were assessed for percentage Annexin V/DAPI positivity by flow cytometry at the indicated time points. (*H*) Transduced and nontransduced populations from *F* were passaged in vitro for 16 d. The percentage of sgRNA^+^ cells (GFP^+^) was assessed by flow cytometry (individual bars represent days 3, 5, 7, 9, 12, 14, and 16 after sgRNA induction). (*I*) MHH-CALL4 cells were transduced with pLMS-sh*JAK2*.209 or pLMS-sh*SCR*. At day 14 after transduction, cell cycle analysis was performed by flow cytometric analysis of BrdU incorporation versus DNA content (7-AAD). Error bars in *D*, *E*, *G*, and *I* represent SEM (*n* = 2); error bars in *H* represent SEM (*n* = 3). (**) *P* < 0.01; (ns) nonsignificant (*P* > 0.05).

We and others demonstrated previously that type I JAK2is can kill JAK2^V617F^-driven MPNs (Supplemental Fig. S1A,B; Geron et al. 2008; Wernig et al. 2008), TEL-JAK2-driven T-cell ALLs (T-ALLs) (Waibel et al. 2013), JAK2-amplified classical Hodgkin lymphomas (cHLs), and primary mediastinal large B-cell lymphomas (Hao et al. 2014). However, these agents had limited activity against mutant JAK2-expressing B-ALL cells, putatively due to paradoxical JAK2^Y1007/1008^ hyperphosphorylation mediated by these agents in these cells (Weigert et al. 2012; Wu et al. 2015). We noted that while ruxolitinib treatment of JAK2^I682F^-expressing MHH-CALL4 B-ALL cells did cause JAK2^Y1007/1008^ hyperphosphorylation (Supplemental Fig. S1B), this agent had little or no effect on cell survival and suppressed cell proliferation only after 6–8 d of continuous culture with drugs (Fig. 1A; Supplemental Fig. S1A). Interestingly, key downstream pathways of mutant JAK2 that regulate cell survival and proliferation such as STATs (STAT1 and STAT5), S6, and MEK/ERK were potently suppressed in ruxolitinib-treated MHH-CALL4 cells to levels equivalent to ruxolitinib-treated JAK2^V617F^-expressing human megakaryoblastic SET-2 cells (Supplemental Fig. S1B). Moreover, the JAK/STAT gene expression signature remained significantly down-regulated following ruxolitinib treatment of MHH-CALL4 cells, indicating the absence of compensatory maintenance of this pathway following JAK2 inhibition with a type I JAK2i (Supplemental Fig. S1C).

These results raised the possibility that the modest effects of type I JAK2is in JAK2 mutant B-ALL may not be due to paradoxical JAK2^Y1007/1008^ hyperphosphorylation but rather may be due to the cells not being primarily reliant on mutant JAK2 for sustained survival and/or proliferation. To test this hypothesis, we transduced MHH-CALL4 cells with retroviral vectors expressing shRNAs targeting JAK2 (sh*JAK2*.209 and sh*JAK2*.2826) or a nontargeting shRNA (sh*SCR*). JAK2 knockdown in MHH-CALL4 cells resulted in decreased phosphorylation of STAT1^Y701^ and STAT5^Y694^ (Fig. 1B) and significant negative enrichment of the JAK/STAT gene expression signature (Fig. 1C), mirroring the biochemical and molecular effects of ruxolitinib. Consistent with the effects of ruxolitinib on B-ALL cell proliferation, JAK2 knockdown did not induce apoptosis (Fig. 1D,I), and competitive growth assays demonstrated a loss of representation of JAK2-depleted MHH-CALL4 cells evident after >8 d (Fig. 1E). In agreement with these findings, CRISPR–Cas9-mediated JAK2 deletion with two independent single guide RNAs (sgRNAs) resulted in little or no induction of cell death (Fig. 1F,G) but led to a loss of representation in a competitive proliferation assay (Fig. 1H). Additional cell cycle analysis of JAK2-depleted MHH-CALL4 cells showed a significant reduction in cells in S phase with a concomitant increase in G_0_/G_1_ populations (Fig. 1I; Supplemental Fig. S1D).

A recent report demonstrated that the type II JAK2i CHZ868, which stabilizes JAK2 in an inactive conformation, kills JAK2 mutant B-ALL cells (Wu et al. 2015). Consistent with that report, we observed potent apoptotic effects of CHZ868 without JAK2^Y1007/1008^ hyperphosphorylation following culture in MHH-CALL4 cells (Supplemental Fig. S1E,F). However, CHZ868 was equipotent in parental MHH-CALL4 cells and those in which JAK2 had been effectively depleted and JAK2 downstream signaling was abrogated (Supplemental Fig. S1F), while ruxolitinib was effective only in JAK2-proficient cells, indicating that the apoptotic effects of CHZ868 were not reliant only on inhibition of JAK2 (Supplemental Fig. S1F). Interestingly, despite their divergent phenotypic response, 3′RNA sequencing (3′RNA-seq) revealed that ruxolitinib did elicit a transcriptional response similar to that of CHZ868 in MHH-CALL4 cells (Supplemental Fig. S1G,H, dimension 1). However, principal component analysis (PCA) did unravel differences in gene expression mediated by ruxolitinib and CHZ868, which may highlight transcriptional changes induced by the JAK2-independent effects of CHZ868 (Supplemental Fig. S1H, dimension 2). Indeed, hierarchical clustering of these genes demonstrated that the ruxolitinib response more closely resembled the transcriptional changes observed following JAK2 knockdown than CHZ868 (Supplemental Fig. S1I). Gene ontology enrichment analysis of the genes altered by CHZ868 and not ruxolitinib or JAK2 depletion revealed enrichment of gene sets related to DNA damage response and DNA replication (Supplemental Fig. S1J). Collectively, these results suggest that the apoptotic effects observed following CHZ868 treatment of MHH-CALL4 B-ALLs are likely due to its JAK2-independent activities, while on-target pharmacological JAK2 inhibition or genetic depletion of JAK2 results in reduced cell proliferation mediated by a delay in transition from G_0_/G_1_ to S phase of the cell cycle (Supplemental Fig. S1D).

### Crlf2 and mutant Jak2 cooperate to induce murine B-ALL development in vivo

**Figure 2.**
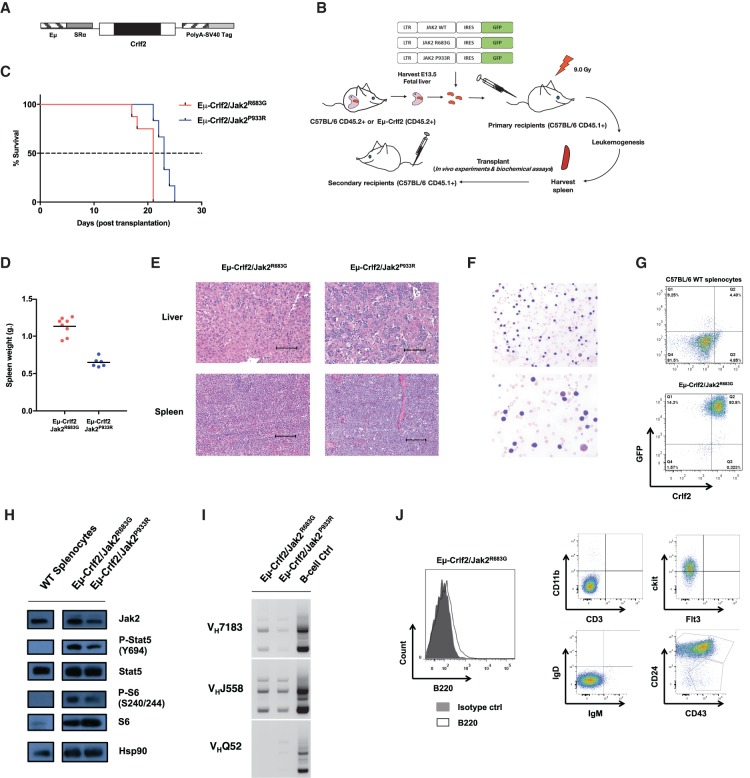
Crlf2 and mutant Jak2 cooperate to induce murine B-ALL development in vivo. (*A*) Schematic of the Eµ-Crlf2 transgene. (*B*) Schematic overview of a fetal liver transplantation mouse model of leukemia. Fetal liver cells isolated at embryonic day 13.5 (E13.5) from Eµ-Crlf2 and wild-type C57BL/6 CD45.2^+^ mice were transduced with pMSCV-IRES-GFP (MIG), MIG-Jak2^WT^, MIG-Jak2^R683G^, or MIG-Jak2^P933R^ and transplanted into sublethally irradiated (9.0 Gy) wild-type C57BL/6 CD45.1^+^ recipients. The resulting primary leukemias were collected and retransplanted into secondary wild-type C57BL/6 CD45.1^+^ recipients to derive a transplantation model for subsequent functional in vivo experiments. (*C*) Kapler-Meier survival curves of cohorts of wild-type C57BL/6 CD45.1^+^ mice transplanted with primary Eμ-Crlf2/Jak2^R683G^ (M#9; *n* = 8) and Eμ-Crlf2/Jak2^P933R^ (M#8; *n* = 6) cells. (*D*) At terminal disease or the conclusion of the experiment, mice in *C* were autopsied, and splenic tumor burden was assessed by weight. (*E*) Hematoxylin–eosin (H&E)-stained spleen and liver sections from moribund wild-type recipient mice transplanted with primary Eμ-Crlf2/Jak2^R683G^ and Eµ*-*Crlf2/Jak2^P933R^ cells. Bars, 100 µm. (*F*) Light microscopy of May-Grunwald-Giemsa-stained peripheral blood smears from moribund secondary recipient mice of Eµ-Crlf2/Jak2^P933R^ cells show marked numbers of medium to large blast cells with high nuclear to cytoplasmic ratio. (*G*) Flow cytometry analysis of Crlf2 and GFP expression on splenocytes isolated from C57BL/6 wild-type mice and moribund secondary recipient mice transplanted with Eμ-*Crlf2*/Jak2^R683G^ cells. A representative flow cytometry plot is shown. (*H*) Immunoblotting was performed against the indicated targets using lysates isolated from nontransformed C57BL/6 wild-type splenocytes, GFP^+^ Eμ-Crlf2/Jak2^R683G^, and Eμ-Crlf2/Jak2^P933R^ cells. Hsp90 served as loading control. (*I*) Nontransformed C57BL/6 wild-type splenic B-cells, GFP^+^ Eμ-Crlf2/Jak2^R683G^, and Eμ-Crlf2/Jak2^P933R^ cells were assayed by PCR of genomic DNA (gDNA) for VDJ rearrangements using the indicated V gene family-specific V_H_ primers. (*J*) Flow cytometric analysis of the GFP^+^ Eμ-Crlf2/Jak2^R683G^ cell population reveals a pre-B-cell immunophenotype (B220^low^IgM^−^IgD^−^CD43^−^CD24^+^ckit^+^CD11b^−^CD3^−^).

The frequent co-occurrence of *CRLF2*-rearrangements and activating JAK2 mutants in B-ALL suggest that these events functionally cooperate in disease pathogenesis (Mullighan et al. 2009a; Harvey et al. 2010; Hertzberg et al. 2010). To more accurately model *CRLF2*-rearranged/JAK2 mutant B-ALL, we initially generated transgenic mice to mimic the recurrent *IGH–CRLF2* and *P2RY8–CRLF2* translocations seen in human B-ALL (Mullighan et al. 2009a; Russell et al. 2009). A transgene consisting of a wild-type *Crlf2* cDNA sequence inserted downstream from the Eµ/SRα promoter/enhancer elements was used to generate transgenic mice with high-level transgene expression (Eµ-Crlf2) exclusively in B-lineage hematopoietic cells (Fig. 2A; Bodrug et al. 1994). Flow cytometric analysis of surface Crlf2 expression confirmed B-cell (B220^+^)-specific high-level transgene expression in the peripheral blood, spleens, and bone marrow of Eµ-Crlf2 transgenic mice (Supplemental Fig. S2A). Eµ-Crlf2-mediated overexpression of Crlf2 alone did not constitutively activate downstream pStat5^Y694^ (Supplemental Fig. S2B), and these mice did not develop B-ALL by 18 mo of age (data not shown), demonstrating that Crlf2 overexpression alone was insufficient to induce B-cell leukemia.

We next assessed the potential leukemogenic cooperation between overexpressed Crlf2 and two distinct B-ALL-associated activating Jak2 point mutants (Jak2^R683G^ or Jak2^P933R^) in vivo. Sublethally irradiated congenic C57BL/6 wild-type CD45.1^+^ recipient mice were transplanted with C57BL/6 wild-type CD45.2^+^ or Eµ-Crlf2 fetal liver cells (FLCs; CD45.2^+^) transduced with retroviral pMSCV-GFP (pMIG), pMIG-Jak2^WT^, pMIG-Jak2^R683G^, or pMIG-Jak2^P933R^ vectors (Fig. 2B). A subset of recipient mice transplanted with Eµ-Crlf2 FLCs expressing Jak2 mutants (Jak2^R683G^ and Jak2^P933R^) succumbed to disease with outgrowth of GFP^+^ populations in the spleen and bone marrow and elevated peripheral white blood cell (WBC) counts and/or splenomegaly (Supplemental Fig. S2C–G). In contrast, mice transplanted with CD45.2^+^ wild-type C57BL/6 FLCs expressing Jak2^WT^ or mutant Jak2 alone or Eµ-Crlf2 FLCs transduced with Jak2^WT^ did not develop any signs of malignancy or outgrowth of GFP^+^ populations by 400 d (Supplemental Fig. S2C–G).

Eμ*-*Crlf2/Jak2^R683G^ (M#9) and Eμ*-*Crlf2/Jak2^P933R^ (M#8) cells harvested from moribund primary recipient mice shown in Supplemental Figure S2C were serially transplantable in secondary recipient C57BL/6 wild-type CD45.1^+^ mice with 100% penetrance (Fig. 2C). Recipient mice succumbed to disease with typical features of hematological malignancy, including hepatosplenomegaly with prominent infiltration of leukemic cells in the spleen and liver (Fig. 2D,E) and the presence of medium to large leukemic blasts with scant cytoplasm and prominent nucleoli in peripheral blood (Fig. 2F). Splenic Eμ*-*Crlf2/mutant Jak2 leukemia cells expressed GFP (surrogate readout of mutant Jak2 expression) and elevated cell surface Crlf2 (Fig. 2G). Moreover, Eμ*-*Crlf2/Jak2^R683G^ (M#9) and Eμ*-*Crlf2/Jak2^P933R^ (M#8) cells demonstrated constitutive pStat5^Y694^ and pS6^S240/244^ (Fig. 2H), which are highly distinctive features of JAK2 mutant B-ALLs among other genetic subtypes (Tasian et al. 2012). PCR analysis of genomic DNA confirmed immunoglobulin heavy chain (IgH; V_H_ to DJ_H_) gene rearrangements in these leukemia cells consistent with B-lineage commitment (Li et al. 1993) and polyclonal IgH rearrangement patterns as described for some cases of human B-ALLs (Fig. 2I; Ford et al. 1983). Consistent with this, flow cytometric analysis revealed that Eμ*-*Crlf2/Jak2 mutant cells expressed a progenitor B-cell immunophenotype (B220^low^CD43^−^CD24^+^ IgM^−^IgD^−^ckit^+^CD3^−^CD11b^−^) (Fig. 2J; Supplemental Fig. S2H). Taken together, these data demonstrate the development of novel mouse models of B-ALL that recapitulate common genetic, biochemical, and pathologic features of human *CRLF2*-rearranged/JAK2 mutant B-ALLs.

### Eµ-Crlf2/Jak2 mutant B-ALLs persist despite sustained Jak2 inhibition or depletion in vivo

**Figure 3.**
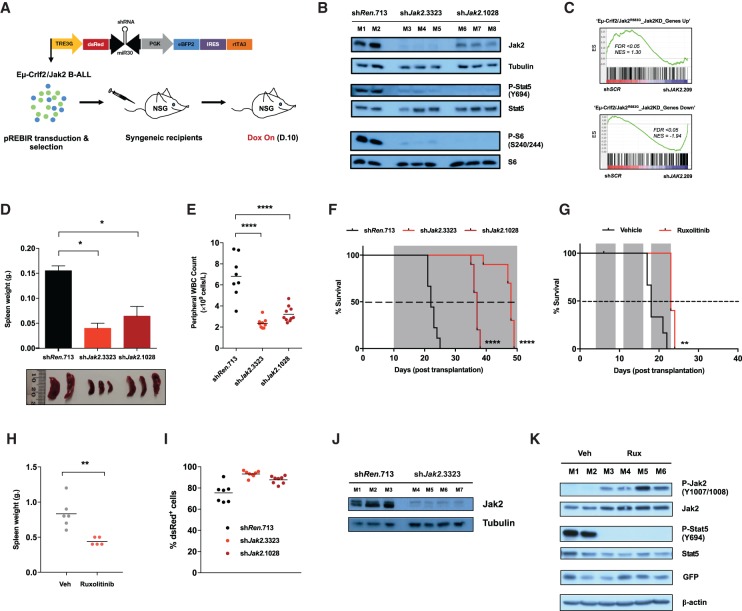
Eµ-Crlf2/Jak2 mutant B-ALLs persist despite sustained Jak2 inhibition or depletion in vivo. (*A*) Schematic representation of the in vivo application of Dox-inducible (Tet-on) pREBIR system. Established Eµ*-*Crlf2/Jak2 mutant B-ALLs were freshly isolated, transduced with pREBIR harboring shRNAs targeting Jak2 (sh*Jak2*.3323 or sh*Jak2*.1028) or sh*Renilla*.713, FACS-sorted for transduced populations (eBFP2^+^), and retransplanted into NSG recipient mice. shRNAs were transiently induced by Dox administration in vivo. (*B*) Cohorts of mice were transplanted with Eµ-Crlf2/Jak2^R683G^-pREBIR-sh*Renilla*.713 (mouse 1 [M1] and mouse 2 [M2]; *n* = 2), Eµ-Crlf2/Jak2^R683G^- pREBIR-sh*Jak2*.3323 (M3–M5; *n* = 3), or Eµ-Crlf2/Jak2^R683G^-pREBIR-sh*Jak2*.1028 (M6–M8; *n* = 3) cells and treated with Dox for 48 h at 10 d after engraftment prior to being sacrificed. Immunoblotting was performed for the indicated targets using whole-cell lysates from dsRed^+^/eBFP2^+^ splenocytes. Tubulin served as a loading control. (*C*) GSEA of JAK2 knockdown human MHH-CALL4 cells at day 5 after transduction (MHH-CALL4-pLMS-sh*SCR* [sh*SCR*] relative to MHH-CALL4-pLMS-sh*JAK2*.209 cells [sh*JAK2*.209]) demonstrates significant enrichment of gene sets induced (“Eµ-Crlf2/Jak2^R683G^_Jak2KD_Genes Down”) and repressed (“Eµ-Crlf2/Jak2^R683G^_Jak2KD_Genes Up”) in Jak2 knockdown mouse Eµ-Crlf2/Jak2^R683G^ B-ALL cells (Eµ-*Crlf2*/Jak2^R683G^-pREBIR-sh*Jak2*.3323 relative to Eµ-Crlf2/Jak2^R683G^-pREBIR-sh*Renilla*.713; 48 h after shRNA induction in vivo). (*D*) Cohorts of recipient mice were transplanted with Eµ-Crlf2/Jak2^R683G^-pREBIR-sh*Renilla*.713 (*n* = 2), Eµ-Crlf2/Jak2^R683G^-pREBIR-sh*Jak2*.3323 (*n* = 3), or Eµ-Crlf2/Jak2^R683G^-pREBIR-sh*Jak2*.1028 (*n* = 3) cells for 10 d and treated with Dox for 48 h prior to being sacrificed and analyzed for spleen weight. Error bars represent SEM. *n* = 2. A photograph of spleens from mice in each treatment arm is shown. The ruler scale is in millimeters. (*E*) Peripheral blood from recipient mice of Eµ-Crlf2/Jak2^R683G^-pREBIR-sh*Renilla*.713 (*n* = 9), Eµ-Crlf2/Jak2^R683G^–pREBIR-sh*Jak2*.3323 (*n* = 10), or Eµ-Crlf2/Jak2^R683G^–pREBIR-sh*Jak2*.1028 (*n* = 10) cells at week 3 after injection was analyzed for total peripheral WBC count. All mice were treated with Dox from day 10 after tumor transplantation. (*F*) Kaplan-Meier survival curve of NSG recipient mice treated as in *D*. Gray shading indicates the period of Dox administration. (*G*) Kaplan-Meier survival curve of recipient mice of Eµ*-*Crlf2/Jak2^R683G^ B-ALL cells treated with vehicle (0.5% methyl cellulose; *n* = 6) or 90 mg/kg ruxolitinib twice per day (*n* = 6) from day 3 after injection by oral gavage. Gray shading indicates the period of drug treatment. (*H*) At terminal disease, mice from *G* were autopsied, and splenic burden was assessed by spleen weight. (*I*) Quantification of dsRed^+^/eBFP2^+^ proportions in GFP^+^ splenocytes collected from moribund bound Dox-treated recipient mice from *F* at terminal disease. (*J*) Western blot analysis was performed for total Jak2 using lysates isolated from dsRed^+^/eBFP2^+^ splenocytes collected from individual Dox-treated moribund recipients of Eµ-Crlf2/Jak2^R683G^-pREBIR-sh*Ren*.713 (M1–M3; *n* = 3) and Eµ-Crlf2/Jak2^R683G^-pREBIR-sh*Jak2*.3323 (M4–M7; *n* = 4) cells from *F* at terminal disease. Tubulin served as a loading control. (*K*) Immunoblotting was performed against the indicated targets using lysates isolated from splenocytes collected from individual vehicle (M1–M2; *n* = 2) or ruxolitinib-treated (M3–M6; *n* = 4) moribund recipients of Eµ-Crlf2/Jak2^R683G^ B-ALLs at terminal disease. β-Actin served as a loading control. (*) *P* < 0.05; (**) *P* < 0.01; (****) *P* < 0.0001.

Using our unique GEMMs of human JAK2 mutant B-ALL, we next assessed the critical importance of Jak2 for leukemia maintenance in vivo. Eµ*-*Crlf2/Jak2^R683G^ cells (GFP^+^) were retrovirally transduced with doxycycline (Dox)-inducible (Tet-on) pREBIR constructs expressing eBFP2 and shRNAs targeting either Jak2 (sh*Jak2*.3323 or sh*Jak2*.1028) or a control nonmammalian gene (sh*Renilla*.713). eBFP2^+^/GFP^+^-sorted cells were transplanted into secondary NOD/SCID IL-2Rγ^−/−^ (NSG) recipient mice (Fig. 3A), and, at day 10 after leukemia transplantation, shRNA expression was induced by Dox administration. Jak2 knockdown in Eµ*-*Crlf2/Jak2^R683G^ cells abrogated pStat5^Y694^ and pS6^S240/244^ (Fig. 3B), and RNA-seq analysis of Eµ*-*Crlf2/Jak2^R683G^ B-ALL with Jak2 knockdown identified gene signatures that were significantly enriched in JAK2-depleted MHH-CALL4 cells, indicating that a similar pattern of transcriptional change is induced upon RNAi-mediated JAK2 depletion across the two model systems (false discovery rate [FDR] < 0.05) (Fig. 3C).

In vivo Jak2 depletion in mice bearing Eµ*-*Crlf2/Jak2^R683G^ leukemias using two different shRNAs resulted in significantly reduced peripheral WBC count, reduced leukemic burden in the spleen (Fig. 3D,E), and a marked survival benefit that correlated with knockdown efficiency of the Jak2 shRNAs (Fig. 3B,F). Similarly*,* knockdown of Jak2 in Eµ*-*Crlf2/Jak2^P933R^ B-ALLs also resulted in enhanced survival of recipient mice, although not to the same extent as seen using Eµ*-*Crlf2/Jak2^R683G^ leukemias (Supplemental Fig. S3A). The therapeutic effects of enzymatic Jak2 inhibition in Eµ*-*Crlf2/Jak2^R683G^ and Eµ*-*Crlf2/Jak2^P933R^ B-ALL cells assessed using JAK2i ruxolitinib also revealed a relatively small but significant survival benefit that corresponded with a reduction in splenic burden at terminal disease, decreased activity of Stat5, and concomitant Jak2^Y1007/1008^ hyperphosphorylation (Fig. 3G,H; Supplemental Fig. S3D–G).

Although in vivo depletion of Jak2 or enzymatic inhibition with ruxolitinib resulted in enhanced survival of mice bearing Eµ*-*Crlf2/mutant Jak2 B-ALLs, all recipient mice that initially responded eventually relapsed and succumbed to disease (Fig. 3F,G; Supplemental Fig. S3A,D,H,I). To determine whether Dox-induced Jak2 knockdown was sustained in these persistent Eµ*-*Crlf2/Jak2^R683G^ cells, eBFP2^+^/GFP^+^ leukemias harvested from the spleens of mice in Figure 3F were analyzed for expression of dsRed as a surrogate readout for shRNA expression. Intriguingly, cells harvested from the spleens of Dox-treated mice transplanted with Eµ*-*Crlf2/Jak2^R683G^-pREBIR-sh*Jak2*.3323 and Eµ*-*Crlf2/Jak2^R683G^-pREBIR-sh*Jak2*.1028 cells retained strong expression of dsRed (Fig. 3I; Supplemental Fig. S3J). Flow cytometric analysis revealed that dsRed^+^ Eµ*-*Crlf2/Jak2^R683G^-pREBIR-sh*Jak2*.3323 cells harvested from moribund Dox-treated mice retained their pre-B-cell immunophenotype (Supplemental Fig. S3K). Subsequent Western blot analysis of these leukemias demonstrated sustained depletion of Jak2 (Fig. 3J, lanes M4–M7), and a decrease in pStat5^Y694^ was observed in dsRed^+^ Eµ*-*Crlf2/Jak2^P933R^-pREBIR-sh*Jak2*.3323 leukemia cells harvested from moribund Dox-treated mice (Supplemental Fig. S3B,C). Similarly, GFP^+^ splenocytes harvested from moribund ruxolitinib-treated Eµ*-*Crlf2/Jak2^R683G^ B-ALL recipient mice retained GFP expression and showed Jak2^Y1007/1008^ hyperphosphorylation and a sustained loss of pStat5^Y694^ (Fig. 3K, M3–M6). These results demonstrate that pharmacological inhibition and genetic depletion of Jak2 in Eµ*-*Crlf2/Jak2 mutant B-ALLs yields a transient therapeutic response in vivo, and Eµ*-*Crlf2/Jak2 mutant B-ALLs with sustained depletion or inhibition of Jak2 and loss of Stat5 signaling remain viable at end-stage disease.

### RNAi-mediated Jak2 depletion induces a partial anti-proliferative response independent of cell death in Eµ-Crlf2/Jak2^R683G^ B-ALLs in vivo

**Figure 4.**
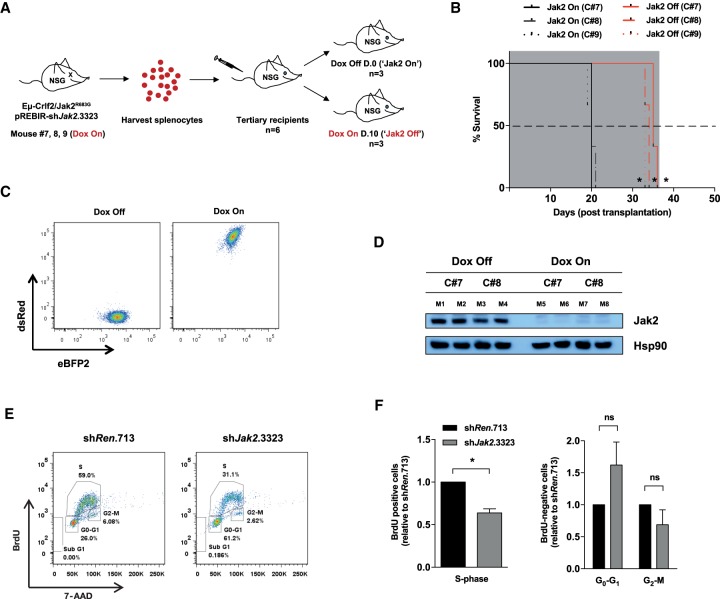
RNAi-mediated Jak2 depletion induces an anti-proliferative response independent of cell death in Eµ-Crlf2/Jak2^R683G^ B-ALLs in vivo. (*A*) Schematic illustration of the experimental design. Splenocytes from moribund bound secondary recipients (mouse 7, mouse 8, and mouse 9 from recipients in Fig. 3F, referred to here as Jak2 knockdown-persistent clone 7 [C#7], clone 8 [C#8], and clone 9 [C#8]) of Eµ-Crlf2/Jak2^R683G^-pREBIR-sh*Jak2*.3323 (Dox on) leukemia cells were harvested and engrafted into tertiary recipient mice. *n* = 6 for each clone. For each clone, mice were left untreated (Dox off; *n* = 3) or treated with Dox (Dox on; *n* = 3) from day 0 after transplantation. (*B*) Kaplan-Meier survival curve of Dox-treated (Jak2 off; *n* = 3 per clone) and untreated (Jak2 on; *n* = 3 per clone) recipient mice of Jak2 knockdown-persistent Eµ-Crlf2/Jak2^R683G^-pREBIR-sh*Jak2*.3323 leukemia cells. Gray shading indicates the period of Dox administration. (*C*) Representative flow cytometric plot showing eBFP2/dsRed proportions of Jak2 knockdown-persistent Eµ-Crlf2/Jak2^R683G^-pREBIR-sh*Jak2*.3323 cells (GFP^+^) in the spleens of Dox-treated (Dox-on) and untreated (Dox-off) recipients in *B* at terminal disease. (*D*) Immunoblotting against the indicated targets was performed using lysates isolated from splenocytes of individual moribund bound Dox-treated (Dox on; eBFP2^+^/dsRed^+^ cells; M5–M8) and untreated (Dox off; eBFP2^+^/dsRed^−^ cells; M1–M4) recipients in *B*. (*E*) Representative flow cytometric plot of BrdU incorporation and 7-AAD staining of eBFP2^+^/dsRed^+^ splenocytes from recipients of Eμ-Crlf2/Jak2^R683G^-pREBIR-sh*Renilla*.713 (sh*Ren*.713) and Eμ-Crlf2/Jak2^R683G^-pREBIR-sh*Jak2*.3323 B-ALLs (sh*Jak2*.3323) at 48 h after in vivo shRNA induction. (*F*) Quantification of S-, G_0_/G_1_-, and G_2_M-phase cell percentages of cell cycle analysis from Figure 6E. Error bars represent SEM (*n* = 2) performed in triplicate (three independent mice). (*) *P* < 0.05; (ns) nonsignificant (*P* > 0.05).

We next sought to determine whether Eµ*-*Crlf2/Jak2^R683G^ leukemias with sustained knockdown of Jak2 (referred to here as “Jak2 knockdown-persistent cells”) obtained from moribund Dox-treated recipient mice (Fig. 3F) maintained an ability to respond to mutant Jak2. Mice were transplanted with Jak2 knockdown-persistent cells (harvested from mouse/clone 7 [C#7], mouse/clone 8, and mouse/clone 9 from Fig. 3F, pREBIR-sh*Jak2*.3323 cohort), and recipient mice were maintained on Dox (“Jak2 off”) or left untreated (“Jak2 on”) from day 0 after transplantation (Fig. 4A).

All Dox-treated (Jak2-off) recipient mice harboring Jak2 knockdown-persistent cells exhibited a delay in leukemia progression and conferred a significant survival benefit over the untreated (Jak2-on) cohort (Fig. 4B). Flow cytometric and Western blot analysis confirmed sustained Jak2 shRNA expression (determined by detection of dsRed) and concomitant depletion of Jak2 in splenic cells harvested from moribund mice in the Dox-on (Jak2-off) group (Fig. 4C,D). In contrast, Jak2 knockdown persistent cells harvested from moribund mice in the Dox-off group re-expressed Jak2 and showed concomitant loss of dsRed expression (Fig. 4C,D), and mice in this cohort succumbed to disease more rapidly than mice in the Dox-treated (Jak2-off) group (Fig. 4B). These data indicate that Eµ*-*Crlf2/Jak2 mutant B-ALLs with sustained knockdown of Jak2 retain their immortality and continue to proliferate, albeit at a reduced rate. Thus, Jak2 expression is not necessary for these cells to persist in vivo.

We next investigated whether Jak2 knockdown invoked changes in cell cycle progression. Eµ*-*Crlf2/Jak2^R683G^-pREBIR-sh*Jak2*.3323 or Eµ*-*Crlf2/Jak2^R683G^-pREBIR-sh*Renilla*.713 cells were established in recipient mice for 10 d prior to Dox-mediated shRNA induction, and cell proliferation was assessed using in vivo BrdU labeling. Consistent with data showing the effects of JAK2 knockdown in MHH-CALL4 cells (Fig. 1I), Jak2-depleted Eµ*-*Crlf2/Jak2^R683G^ B-ALLs demonstrated a significant reduction in the proportion of leukemic cells in S phase (Fig. 4E,F).

### Genome-wide transcriptome analysis of Jak2 knockdown persistent Eμ-Crlf2/Jak2^R683G^-driven B-ALLs

**Figure 5.**
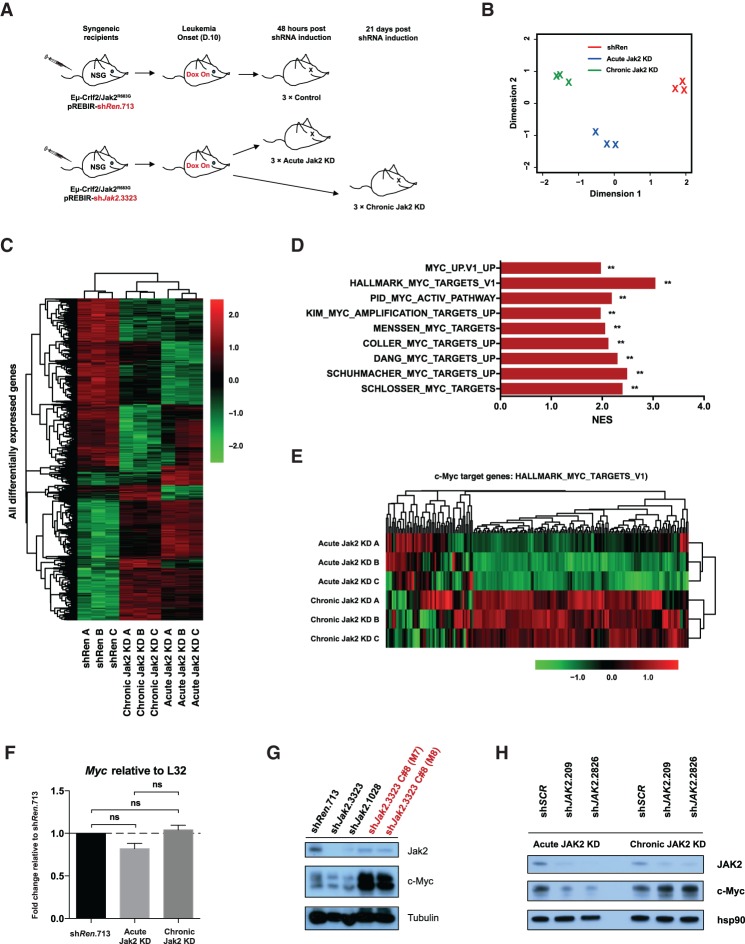
Genome-wide transcriptome analysis of Jak2 knockdown-persistent Eμ-Crlf2/Jak2^R683G^-driven B-ALLs. (*A*) Schematic illustration of the generation of samples for RNA-seq. Dox-treated (day 10 after transplantation) recipient mice of Eµ-Crlf2/Jak2^R683G^-pREBIR-sh*Renilla*.713 (*n* = 3) or Eµ-Crlf2/Jak2^R683G^–pREBIR-sh*Jak2*.3323 cells (*n* = 6) were sacrificed at either 48 h (acute knockdown) or 21 d (chronic knockdown) after shRNA induction, and RNA-seq was performed using RNA isolated from dsRed^+^/eBFP2^+^ splenocytes. (*B*) PCA (dimension 1 vs. dimension 2) of the top 500 most differentially expressed genes between naive (sh*Ren*; *n* = 3), acute Jak2 knockdown (*n* = 3), and chronic Jak2 knockdown (*n* = 3) Eµ-Crlf2/Jak2^R683G^ B-ALLs. (*C*) Heat map of all significantly differentially expressed genes in all samples subjected to RNA-seq. Adjusted *P* < 0.05; log_2_ fold Δ ≥1 and −1 or less. The scale bar represents fold expression changes of each gene relative to the average gene expression across all samples. (*D*) Summary of normalized enrichment score values from GSEA performed on multiple independent established MYC gene signatures using genes differentially expressed between chronic and acute Jak2 knockdown Eμ-Crlf2/Jak2^R683G^ B-ALLs. (**) FDR < 0.05. (*E*) Heat map of c-Myc target gene expression (HALLMARK_MYC_TARGETS_V1) in Eμ-Crlf2/Jak2^R683G^-driven B-ALL cells subjected to acute (48 h; acute Jak2 knockdown A–C) or chronic Jak2 knockdown in vivo (21 d; chronic Jak2 knockdown A–C). The scale bar represents fold expression changes of each gene relative to the average gene expression across all samples. (*F*) Quantitative PCR (qPCR) was performed using the RNA samples used for RNA-seq in *A* to determine relative mRNA levels of *Myc* in Eµ*-*Crlf2/Jak2^R683G^ B-ALLs subjected to pREBIR-induced acute sh*Renilla*.713, acute sh*Jak2*.3323 (acute Jak2 knockdown), or chronic sh*Jak2*.3323 (chronic Jak2 knockdown) expression in vivo. Error bars represent SEM. *n* = 2. (ns) Nonsignificant (*P* > 0.05). (*G*) Immunoblotting against the indicated targets was performed on lysates isolated from dsRed^+^/eBFP2^+^ splenocytes from mice transplanted with Eµ-Crlf2/Jak2^R683G^-pREBIR-sh*Renilla*.713 (sh*Ren*.713), Eµ-Crlf2/Jak2^R683G^-pREBIR-sh*Jak2*.3323 (sh*Jak2*.3323), and Eµ-Crlf2/Jak2^R683G^-pREBIR-sh*Jak2*.1028 cells (sh*Jak2*.1028) at 48 h after shRNA induction and moribund bound Dox-treated tertiary recipients (sh*Jak2*.3323 C#8 [M7] and sh*Jak2*.3323 C#8 [M8]; red) of Jak2 knockdown-persistent cells (C#8) in Figure 4B. Tubulin served as a loading control. (*H*) Immunoblotting for the indicated targets was performed on lysates from GFP^+^ MHH-CALL4-pLMS-sh*SCR* (sh*SCR*), MHH-CALL4-pLMS-sh*JAK2*.209 (sh*JAK2*.209), and MHH-CALL4-pLMS-sh*JAK2*.2826 (sh*JAK2*.2826) cells at day 5 (acute JAK2 knockdown) and day 12 (chronic JAK2 knockdown) after transduction. Hsp90 served as a loading control.

We hypothesized that Eµ*-Crlf2*/Jak2 mutant B-ALLs that maintained proliferative capacity following sustained (chronic) Jak2 depletion developed compensatory cell growth mechanisms that would be apparent when comparing gene expression signatures in these cells with parental cells and cells subjected to only short-term (acute) Jak2 depletion. RNA-seq was performed on Eµ*-*Crlf2/Jak2^R683G^ B-ALLs that were subjected to 2 d (acute Jak2 knockdown) or 21 d (chronic Jak2 knockdown) of RNAi-mediated Jak2 knockdown in vivo (Fig. 5A). Consistent with our hypothesis, global transcriptome analysis using two distinct methodologies (PCA and unsupervised hierarchical clustering of all differentially expressed genes) confirmed that naïve Eµ*-*Crlf2/Jak2^R683G^ B-ALL cells (control) and those with acute or chronic knockdown of Jak2 all displayed distinct transcriptional profiles, and their respective replicates clustered identically (Fig. 5B,C).

To identify putative “adaptive pathways” apparent in Eµ*-*Crlf2/Jak2^R683G^ leukemia cells with chronic relative to acute Jak2 depletion, gene set enrichment analysis (GSEA) was performed against all publicly available oncogenic signature data sets (C6, Broad Institute Molecular Signatures Database [MSigDB]) (Subramanian et al. 2005). Strikingly, a significant positive enrichment for the MYC gene signature was apparent in Eµ*-*Crlf2/Jak2^R683G^ leukemia cells with chronic Jak2 depletion across multiple data sets (FDR < 0.01) (Fig. 5D; Supplemental Fig. S4A). In addition, GSEA demonstrated up-regulation of multiple gene sets containing c-Myc-binding sites (Supplemental Fig. S4B). There was no up-regulation of gene sets containing STAT5A-binding sites (Supplemental Fig. S4B), indicating that Eµ*-*Crlf2/Jak2^R683G^ B-ALLs that persisted despite sustained depletion of Jak2 did not do so by reactivating STAT5A target genes. Analysis of enriched motifs in promoters of genes induced upon chronic relative to acute Jak2 depletion revealed c-Myc as the top-ranked transcription factor (*P* < 0.001) (Supplemental Fig. S4C). Finally, transcriptome-wide analysis using a larger c-Myc target gene set (HALLMARK_MYC_TARGETS_V1) revealed that the expression of a large number of these genes displayed enhanced expression in Eµ*-*Crlf2/Jak2^R683G^ B-ALL cells with sustained (chronic) Jak2 depletion (Fig. 5E).

Myc transcript levels in chronic Jak2 knockdown Eµ*-*Crlf2/Jak2^R683G^ cells were not significantly elevated relative to acute Jak2 knockdown cells (Fig. 5F). However, consistent with the apparent global up-regulation of Myc target genes in chronic Jak2 knockdown Eµ*-*Crlf2/Jak2^R683G^ cells, c-Myc protein levels were prominently up-regulated in Eµ*-*Crlf2/Jak2^R683G^ leukemia cells following sustained Jak2 depletion (sh*Jak2*.3323, C#8 M7 and M8) (Fig. 5G). This indicates that enhanced signaling through c-Myc in Eµ*-*Crlf2/Jak2^R683G^ leukemia cells following chronic Jak2 depletion is mediated by enhanced c-Myc protein levels in the absence of any increase in mRNA levels. Myc expression remained unchanged when inhibition of Jak2 was relieved in tertiary recipients of chronic Jak2 knockdown Eµ*-*Crlf2/Jak2^R683G^ cells (Supplemental Fig. S4D) despite mice in this cohort succumbing to disease more rapidly than mice in which Jak2 knockdown was maintained (Fig. 4B).

We next assessed whether JAK2 mutant human B-ALLs exhibited similar compensatory proliferative mechanisms following prolonged JAK2 depletion. MHH-CALL4 cells were transduced with pLMS-GFP vectors expressing control (sh*SCR*) or JAK2 targeting (sh*JAK2*.209 or sh*JAK2*.2826) shRNAs, and cells were harvested at day 4 (acute JAK2 knockdown) or day 10 (chronic JAK2 knockdown) for immunoblotting analysis. MHH-CALL4 cells with acute depletion of JAK2 using two independent *JAK2* shRNAs had reduced c-Myc expression (Fig. 5H). However, c-Myc expression was prominently up-regulated following chronic JAK2 knockdown (Fig. 5H), similar to the result seen in Eµ*-*Crlf2/Jak2^R683G^ B-ALLs, demonstrating similar compensatory proliferative mechanisms across the two models. Taken together, these data demonstrate up-regulation of c-Myc and global amplification of its target genes following sustained Jak2 knockdown in B-ALLs driven by oncogenic Jak2.

### Combined targeting of JAK2 and c-Myc is effective against CRLF2/JAK2 mutant B-ALLs

**Figure 6.**
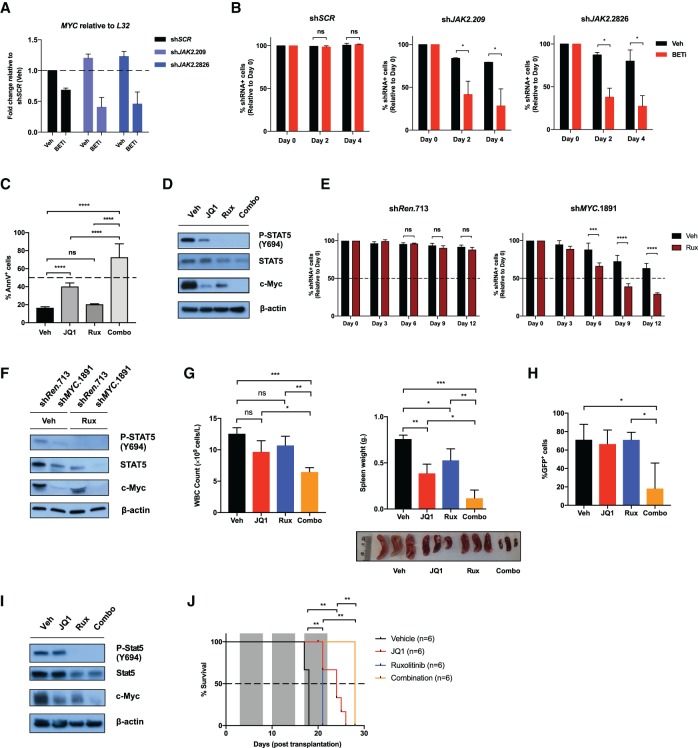
Combined targeting of JAK2 and c-Myc is effective against CRLF2/JAK2 mutant B-ALLs. (*A*) MHH-CALL4-pLMS-sh*SCR*, MHH-CALL4-pLMS-sh*JAK2*.209, and MHH-CALL4-pLMS-sh*JAK2*.2826 cells were treated with vehicle (Veh; DMSO) or 500 nM iBET151 (BETi) for 24 h at day 12 after transduction, and mRNA levels for *MYC* were determined by qPCR. (*B*) Cells in *A* were analyzed for the percentage of shRNA-expressing cells (GFP^+^) by flow cytometry (days 0, 2, and 4 after drug treatment), and data were normalized to day 0. (*C*) MHH-CALL4 cells were exposed to vehicle (Veh; DMSO), 500 nM JQ1, 1000 nM ruxolitinib (Rux), or the combination (Combo) for 72 h, and the percentage of Annexin V-positive (AnnV^+^) cells was assessed. (*D*) Immunoblotting for the indicated targets was performed on lysates from MHH-CALL4 cells treated as in *C* for 24 h. (*E*) MHH-CALL4 cells were transduced with constitutive (pLMS-eBFP2) retroviral vectors harboring sh*MYC*.1891 or sh*Renilla*.713 (sh*Ren*.713). At day 5 after transduction, transduced and nontransduced populations were passaged in vitro for 12 d with vehicle (Veh; DMSO) or 1000 nM ruxolitinib (Rux). Drugs were replenished to the indicated concentrations, and the percentage of eBFP2^+^ cells was assessed by flow cytometry every 3 d. (*F*) Immunoblotting for the indicated targets was performed against MHH-CALL4-pLMS-sh*Renilla*.713 (sh*Ren*.713) and MHH-CALL4-pLMS-sh*MYC*.1891 (sh*MYC*.1891) cells treated with vehicle (Veh; DMSO) or 1000 nM ruxolitinib (Rux) for 24 h. (*G*, *H*) Cohorts of C57BL/6 wild-type CD45.1^+^ mice were transplanted with Eµ*-*Crlf2/Jak2^R683G^ B-ALL. After 12 d, mice were treated with vehicle (Veh), 50 mg/kg JQ1 once per day, 90 mg/kg ruxolitinib (Rux) twice per day, or the combination (Combo; JQ1 and ruxolitinib). Spleen weight, total peripheral WBC count, and percentage of GFP^+^ cells in the spleens of individual mice in each treatment group were assessed after 3 d of therapy. A photograph of spleens from mice in each treatment arm is shown. *n* = 3 per group. The ruler scale is in centimeters. (*I*) Immunoblotting for the indicated targets was performed on lysates from Eμ-Crlf2/Jak2^R683G^ B-ALLs treated with vehicle (Veh; DMSO), 500 nM JQ1, 1000 nM ruxolitinib (Rux), or the combination ex vivo for 8 h. (*J*) Kaplan-Meier survival curve of recipient mice transplanted with Eµ*-*Crlf2/Jak2^R683G^ B-ALLs treated as in *G* from 3 d after transplantation (the gray area indicates the period of drug treatment). β-Actin served as a loading control for all immunoblots. Error bars in *A–C* represent SEM (*n* = 2); error bars in *E* represent SEM (*n* = 3). (*) *P* < 0.05; (**) *P* < 0.01; (***) *P* < 0.001; (****) *P* < 0.0001; (ns) nonsignificant.

Small molecule BET bromodomain inhibitors (BETis) have been shown previously to suppress c-Myc mRNA and expression of its target genes in a range of tumors, including JAK2 mutant B-ALLs (Dawson et al. 2011; Delmore et al. 2011; Zuber et al. 2011b; Ott et al. 2012). MHH-CALL4 cells expressing control shRNA (sh*SCR*) or two JAK2 targeting shRNAs (sh*JAK2*.209, sh*JAK2*.2826) were harvested and cultured with BETis after 10 d of culture when knockdown of JAK2 was evident (Supplemental Fig. S5A). Interestingly, while *MYC* levels were equivalently suppressed in all three cell lines (Fig. 6A), only MHH-CALL4-pLMS-sh*JAK2*.209 and MHH-CALL4-pLMS-sh*JAK2*.2826 cells with chronic (10-d) depletion of JAK2 demonstrated sensitivity to BETi-induced apoptosis (Fig. 6B). These results demonstrate that MHH-CALL4 cells with sustained depletion of JAK2 demonstrate a synthetic-lethal response to BETi-mediated down-regulation of *MYC*. The potent effects of dual targeting of JAK2 and c-Myc in MHH-CALL4 cells were further demonstrated using pharmacological agents ruxolitinib and JQ1 (Fig. 6C). In addition to the enhanced apoptotic response to the combination of ruxolitinib and JQ1, these agents provided superior down-regulation of c-Myc when combined compared with either single agent (Fig. 6D). Congruent with these findings, chronic c-Myc-depleted MHH-CALL4-pLMS-sh*MYC*.1891 cells demonstrated enhanced sensitivity to ruxolitinib relative to MHH-CALL4 cells expressing the control shRNA (Fig. 6E), and the combination of ruxolitinib and c-Myc knockdown induced superior down-regulation of c-Myc compared with either knockdown or ruxolitinib alone (Fig. 6F; Supplemental Fig. S5B). Intriguingly, c-Myc knockdown in MHH-CALL4 cells resulted in reduced phosphorylation and expression of STAT5 (Fig. 6F) reminiscent of JQ1 treatment, which down-regulates surface IL-7R expression and leads to reduced STAT5^Y694^ phosphorylation in this context (Fig. 6D; Ott et al. 2012). However, flow cytometric analysis revealed no significant differences in surface expression of either IL-7R or CRLF2 in MHH-CALL4-pLMS-sh*MYC*.1891 cells relative to MHH-CALL4-pLMS-sh*Renilla*.713 cells (Supplemental Fig. S5C).

To assess the in vivo potential of concurrent JAK2 and c-Myc inhibition in vivo, mice were transplanted with Eµ*-*Crlf2/Jak2^R683G^ B-ALL cells and treated with 50 mg/kg JQ1 once per day and 90 mg/kg ruxolitinib twice per day alone or in combination. After 3 d of drug treatment, mice receiving the combination had significantly lower peripheral WBC count, marked reductions in splenomegaly, and reduced splenic tumor burden (assessed by percentage GFP positivity) compared with mice treated with vehicle or either single agent (Fig. 6G,H). The biochemical effects seen in MHH-CALL4 cells (Fig. 6D) following single-agent and combination treatment with JQ1 and ruxolitinib were also observed in Eµ*-*Crlf2/Jak2^R683G^ B-ALL cells following in vivo treatment with these agents (Fig. 6I), highlighted by the almost complete loss of c-Myc following combination treatment. Additional cohorts of mice transplanted with Eµ*-*Crlf2/Jak2^R683G^ B-ALL cells were treated for 3 wk with JQ1 and/or ruxolitinib. Both single-agent ruxolitinib and JQ1 increased overall survival compared with vehicle treatment (Fig. 6J), while the combination therapy, which was well-tolerated (Supplemental Fig. S5D–F), further prolonged overall survival, with an improvement in median survival from 18 d in vehicle-treated mice to 28 d in mice following combination treatment (Fig. 6J).

## Discussion

The discovery of mutant constitutively active JAK2 in high-risk B-ALL highlighted JAK2 as a promising novel therapeutic target in this disease. However, preclinical studies showed that type I JAK2is induce persistent JAK2^Y1007/1008^ hyperphosphorylation and exhibit limited efficacy against JAK2 mutant B-ALLs (Weigert et al. 2012; Wu et al. 2015). A phase II clinical trial is currently under way testing ruxolitinib in CRLF2/JAK2 mutant Ph-like B-ALL, further highlighting the critical need to elucidate the relevance of JAK2 as a therapeutic target in this disease (ClinicalTrials.gov identifier: NCT02723994).

Here, we developed novel GEMMs of human B-ALL through transgenic overexpression of Crlf2 and concomitant expression of mutant Jak2 to determine the role of mutant JAK2 in leukemia initiation and maintenance of disease. We initially showed that Crlf2 and mutant Jak2 alone (Jak2^R683G^ and Jak2^P933R^) were insufficient for B-ALL induction using the fetal liver transplantation approach, congruent with previous findings using transgenic Eµ*-*JAK2^R683G^ mouse models (Lane et al. 2014). Moreover, we demonstrated that while mutant Jak2 can cooperate with Crlf2 to drive development of leukemia in mice, genetic depletion or pharmacological inhibition of Jak2 served merely to delay proliferation of the leukemias, resulting in a modest therapeutic response in vivo. CRLF2/JAK2 mutant B-ALL cells that remained viable following long-term depletion or pharmacological inhibition of JAK2 developed an adaptive response that featured enhanced expression of c-Myc and downstream Myc target genes. Importantly, this adaptive response revealed a synthetic lethality to both RNAi- and BETi-associated down-regulation of c-Myc expression, forming the rationale for combining BETis with JAK2i ruxolitinib, which proved to be a superior therapeutic strategy in vitro and in vivo compared with either single-agent treatment.

Our study challenges the notion that type I JAK2is such as ruxolitinib are relatively ineffective for the treatment of CRLF2/JAK2 mutant leukemia due to paradoxical JAK2^Y1007/1008^ hyperphosphorylation (Weigert et al. 2012; Wu et al. 2015). While our new data and previously published reports (Weigert et al. 2012; Wu et al. 2015) clearly demonstrate that ruxolitinib does induce JAK2 hyperphosphorylation in JAK2 mutant B-ALLs, it is also clear that key downstream targets of JAK2 such as STAT1, STAT3, STAT5, S6, and ERK remain in an inactive state with suppression of the STAT5-dependent gene expression signature. While the type II JAK2i CHZ868 did effectively kill CRLF2/JAK2 mutant B-ALL cells without inducing JAK2^Y1007/1008^ hyperphosphorylation, this agent remained effective even in cells in which the putative molecular target (JAK2) had been genetically depleted. This therefore raises questions regarding the off-target effects of the CHZ868 type II inhibitor that require further comprehensive evaluation and more broadly about the importance of mutant JAK2 in maintaining cell proliferation or survival in JAK2 mutant B-ALLs.

Using both established human CRLF2/JAK2 mutant B-ALL cells and primary mouse Eµ*-*Crlf2/Jak2^R683G^ or Eµ*-*Crlf2/Jak2^P933R^ leukemias, we showed that depletion or inhibition of Jak2 did not affect the viability of these cells but resulted in fewer leukemic cells entering into the cell cycle, which was reflected by a modest therapeutic response in vivo. Our findings were congruent with various other studies demonstrating minimal efficacy of single-agent ruxolitinib in multiple independent JAK2 mutant ALL patient-derived xenograft (PDX) models and the lack of cytotoxicity observed in JAK2 mutant human cell lines performed in short-term in vitro assays (Maude et al. 2012; Tasian et al. 2012; Weigert et al. 2012; Steeghs et al. 2017). Eµ*-*Crlf2/Jak2 mutant B-ALL cells that had remained viable even weeks after knockdown of Jak2 in vivo retained leukemogenic potential and remained partially sensitive to Jak2 depletion, as shown by serial transplant experiments. However, when Jak2 expression was relieved in these cells, the leukemias were more aggressive, indicating that oncogenic signaling pathways downstream from mutant Jak2 remained intact in these cells.

How, then, do CRLF2/JAK2 mutant B-ALL cells that depend on mutant JAK2 for disease initiation remain alive and proliferating (albeit more slowly) following sustained pharmacological inhibition or genetic depletion of the driving oncoprotein JAK2? We have no biochemical or molecular evidence to suggest that JAK/STAT signaling had been restored in leukemias with sustained inhibition or depletion of JAK2. This is in contrast to studies using JAK2^V617F^ mutant SET-2 megakaryoblastic leukemia cells that acquire resistance following prolonged culture in ruxolitinib (Koppikar et al. 2012). In that system, ruxolitinib-resistant SET-2 cells maintained JAK/STAT signaling through compensatory heterodimeric activation of JAK2 by other JAK family members such as JAK1 and TYK2, and RNAi-mediated depletion of JAK2 resulted in decreased viability of the resistant populations (Koppikar et al. 2012). Interestingly, it is apparent that SET-2 myeloproliferative disorder-derived megakaryoblastic leukemia cells expressing JAK2^V617F^ undergo rapid ruxolitinib-mediated apoptosis (Geron et al. 2008; Wernig et al. 2008) as opposed to the mild suppression in cell growth seen in ruxolitinib-treated CRLF2/JAK2 mutant B-ALL cells that we demonstrated here. The difference in biological response to ruxolitinib seen on the different JAK2 mutant cells occurs even though ruxolitinib equivalently suppressed phosphorylation of STAT1, STAT5, MEK/ERK, and S6 and induced JAK2^Y1007/1008^ hyperphosphorylation in all cells (Supplemental Fig. S1B). These data indicate that JAK2^Y1007/1008^ hyperphosphorylation is not a reliable biomarker for response to ruxolitinib in preclinical models and that dephosphorylation of downstream JAK2 targets, including STATs, MEK/ERK, and S6, is not sufficient to potently kill all JAK2 mutant malignancies.

Our study provides novel molecular insights into how JAK2 mutant B-ALL cells adapt to depletion or inhibition of JAK2 to maintain proliferative and leukemic potential. We discovered positive enrichment of the Myc gene expression signature and up-regulation of c-Myc protein expression in CRLF2/JAK2 mutant B-ALL cells subjected to prolonged JAK2 depletion. At present, it is unclear how c-Myc expression is enhanced in CRLF2/JAK2 mutant B-ALL cells with sustained depletion/inhibition of JAK2; this remains to be elucidated in future studies, but the response did not appear to be driven by transcriptional up-regulation of c-Myc expression. It is possible that post-translational modifications of c-Myc, including phosphorylation, acetylation, and/or ubiquitination (Li et al. 2001; Adhikary and Eilers 2005; Faiola et al. 2005), may have contributed to its increased expression in CRLF2/JAK2 mutant B-ALL cells with sustained depletion or inhibition of JAK2 through one or more of the various signal transduction pathways that are known to regulate c-Myc expression (Vervoorts et al. 2006). Interestingly, we observed further interplay between c-Myc and the JAK/STAT signaling pathway in which phosphorylation and expression of STAT5 were suppressed upon c-Myc knockdown in MHH-CALL4 cells. We hypothesized that this may be due to the modulation of surface CRLF2 and/or IL-7R expression, reminiscent of prior studies demonstrating JQ1-induced IL-7R down-regulation and concomitant suppression of pSTAT5 (Ott et al. 2012). However, our data suggest that this association is independent of either surface CRLF2 or IL-7R expression modulation, thus warranting further investigations to elucidate the precise mechanism underlying this intriguing interplay between c-Myc and the JAK/STAT pathway in JAK2 mutant B-ALLs.

Interestingly, CRLF2/JAK2 mutant B-ALL cells with sustained depletion/inhibition of JAK2 generated a “neo-dependency” on c-Myc signaling, shown through the enhanced sensitivity of these cells to both RNAi-mediated depletion of c-Myc and the BET bromodomain inhibitors iBET151 and JQ1. Consistent with a previous report using a xenograft model of *CRLF2*-rearranged B-ALL, JQ1 provided a survival advantage to mice transplanted with syngeneic CRLF2/JAK2 mutant B-ALLs (Ott et al. 2012); however, this effect was augmented through coadministration of JAK2i ruxolitinib, which together demonstrated potent repression of c-Myc and induction of apoptosis in vitro. These results provide evidence that this well-tolerated combination approach may be an effective therapeutic strategy to treat JAK2 mutant B-ALLs and thus provide impetus for further preclinical and clinical testing of this combinatorial regimen.

## Materials and methods

### Cell culture

Cell lines were cultured as described previously (Waibel et al. 2013). For short-term ex vivo experiments, Eµ-Crlf2/Jak2 mutant cells were cultured in Anne Kelso-modified Dulbecco's modified Eagle medium (Peter MacCallum Cancer Centre [PMCC], Research Division, Media Kitchen) supplemented with 20% FCS, 100 µM L-asparagine (Sigma-Aldrich), 100 U/mL penicillin (Invitrogen), and 100 mg/mL streptomycin (Invitrogen) at 37°C under 10% CO_2_.

### Compounds

Ruxolitinib and CHZ868 were provided by Novartis. (+)-JQ1 (JQ1) and iBET-151 were kindly provided by James Bradner (Dana Farber Cancer Institute, Boston, MA). For in vitro use, compounds were dissolved in dimethylsulfoxide (DMSO) (Sigma-Aldrich) at a final stock concentration of 10 mM (stored at −20°C) and diluted to the appropriate concentration from stock prior to use. For in vivo application, ruxolitinib was dissolved and administered orally (oral gavage) in 0.5% methylcellulose solution (Sigma-Aldrich) at 90 mg/kg twice daily for the indicated duration. JQ1 was reconstituted in one part DMSO to nine parts 10% (w/v) hydroxypropyl-β-cyclodextrin (HPBCD) (Sigma-Aldrich) solution and administered via intraperitoneal injection at 50 mg/kg once daily for the indicated duration.

### Western blotting

Western blot analysis of whole-cell lysates from frozen or freshly processed cell pellets was performed as described previously (Waibel et al. 2013) using primary antibodies against pJAK2^Y1007/1008^ (3776), JAK2 (3230), pSTAT5^Y694^ (9351), STAT5 (9363), pSTAT1^Y701^ (7649S), pS6^S240/244^ (2215S), S6 (2317), pERK^T202/Y204^ (9101), ERK (9107), c-Myc (Cell Signaling Technology, 9402), STAT1 (BD Biosciences, 610185), GFP (Invitrogen, A6455), 200 µL of β-actin (Sigma-Aldrich. A2228), Hsp90 (Enzo, ADI-SPA-830), and tubulin (Sigma-Aldrich, T5168). Immunoreactive bands were revealed using ECL reagents (Amersham ECL or ECL Prime, GE Healthcare) by film exposure (Fujifilm Super RX, Fujifilm) using an Agfa CP1000 developer (Agfa).

### In vivo mouse experimentation

All experimental mice used in this study were housed in the animal facility at the PMCC (Melbourne, Australia). All procedures were conducted according to the PMCC Animal Experimental Ethics Committee guidelines (animal ethics application approval number E533) and in compliance with the NHMRC (National Health and Medical Research Council) “Australian Code of Practice for the Care and Use of Animals for Scientific Purposes.” For transplantation of Eµ-Crlf2/Jak2 mutant B-ALLs in vivo, cohorts of 6- to 8-wk-old male NSG or C57BL/6 CD45.1^+^ wild-type mice were inoculated via tail vein injection with 2.5 × 10^5^ to 5.0 × 10^5^ Eµ-Crlf2/Jak2 mutant cells (in PBS). For shRNA induction in vivo, mice received Dox-supplemented drinking water (2 mg/mL with 2% sucrose; Sigma-Aldrich) and 625 mg/kg food (Specialty Feeds) for the specified time points. Transplanted mice were monitored weekly for leukemia development by weekly analysis of circulating leukemia cells (by flow cytometry) and assessment of total WBCs (Sysmex hematology analyzer, Sysmex Corporation) from peripheral blood. In all in vivo experiments, moribund bound mice were determined by animal technicians that were blinded of the treatment groups. For a detailed description of the fetal liver transplantation model, please see the Supplemental Material.

### Cell viability and proliferation assays

For cell viability assays, cells were resuspended in 150 µL of Annexin V-binding buffer (10.0 mM HEPES at pH 7.4, 140.0 mM NaCl, 5.0 mM CaCl_2_ in dH_2_O) containing 1:100 APC-conjugated Annexin V (BD Pharmingen), 1.0 µg/mL propidium iodide (Sigma-Aldrich), and/or 1.0 µg/mL DAPI (Sigma-Aldrich) and analyzed by flow cytometry in a 96-well plate (BD FACSVerse). Total viable cell counts were calculated using viable cell concentration data (volumetric cell counting). For proliferation assays, MHH-CALL4 cells were stained with CellTrace Violet (CTV) (Thermo Fisher) as per the manufacturer's instructions, FACS-sorted to a single population (BD FACS Aria III) of CTV^+^ cells, and assessed for CTV expression every 48 h by flow cytometry (BD LSR II flow cytometer). For details on BrdU/7-AAD cell cycle analysis, see the Supplemental Material.

### Retroviral transduction and competitive proliferation assays

Retroviral pLMS-GFP/eBFP2 or pREBIR-eBFP2 (see the Supplemental Material for details on plasmid and shRNA sequence design) RNAi constructs were cotransfected with an appropriate packaging vector (pCL-Eco or pCL-Ampho) into HEK293T cells using calcium phosphate as described previously (Newbold et al. 2013). Viral supernatants were centrifuged onto RetroNectin-coated (30 µg per well; Takara, Clontech) six-well plates followed by spinfection of leukemia cells. The transduction efficiency in MHH-CALL4 cells was assessed 72 h after infection by flow cytometry (GFP or eBFP2 expression), and competitive proliferation assay was performed as described previously (Zuber et al. 2011a). For in vivo shRNA-mediated knockdown studies, Eμ-Crlf2/Jak2 mutant cells were injected intravenously into NSG mice 8 h after retroviral transduction. Established tumors were harvested, and eBFP2-positive cells were isolated by flow cytometry and retransplanted into NSG recipients by intravenous injection to obtain a pure population of transduced cells for experimentation.

### CRISPR–Cas9-mediated gene knockout

sgRNA oligonucleotides (Sigma-Aldrich) targeting *JAK2* were annealed and cloned into lentiviral expression vector FgH1tUTG (Addgene, 70183; a gift from M. Herold) for inducible sgRNA expression (see the Supplemental Material for sgRNA sequences). Cells were first transduced with the Cas9 expression vector FUCas9Cherry (Aubrey et al. 2015), selected for mCherry-positive cells, and subsequently transduced with a lentiviral sgRNA expression vector. Cells were treated with 1.0 µg/mL Dox for transient sgRNA expression.

### Immunophenotyping analysis

Single-cell suspension of bone marrow was obtained by mechanical dissociation using mortar and pestle, and whole spleens were mashed into suspension using a 70-µm cell strainer (BD Falcon). Bone marrow cells, splenocytes, and peripheral blood from mice were red cell-lysed in ACK lysis buffer, and cells were incubated with cell surface FACS antibodies (in FACS buffer; 2% FCS in PBS) for at least 1 h on ice before analysis on a BD LSR II or BD LSR Fortessa flow cytometer. For staining with antibody cocktails, compensation was set using single-stained controls. B220-APCCy7 (103223), B220-APC (103211), and Flt3-PE (135306) were purchased from Biolegend. CD3-PE (561799), Mac1-PerCPCy5.5 (550993), IgD-PE (558597), IgM-APC (550676), CD19-PECy7 (552854), CD24-PerCPCy5.5 (562360), and CD43-PECy7 (562866) were purchased from BD Biosciences. ckit-PerCP eFluor710 (46-1171-80), hTSLPR (CRLF2)-PE (12-5499-42), and hCD127-PECy7 (25-1278-41) were purchased from eBioscience. mTSLPR (Crlf2)-PE (FAB5461P) was purchased from R&D Systems. An IgG isotype antibody was used as a negative control for IL7R (IgG1-PECy7; BD Biosciences, 557872) and CRLF2 (IgG2a-PE; eBioscience, 12-4724-82).

### Histology

Leukemia-infiltrated livers and spleens from mice were resected and fixed in 10% neutral-buffered formalin (NBF) for at least 24 h prior to embedding tissue blocks in paraffin. Tissue processing, embedding, paraffin sectioning of tissues, and staining (hematoxylin and eosin; H&E) were performed by the Advanced Histology Platform (PMCC). All images were captured using the BX61 microscope (Olympus) at the Advanced Histology Platform (PMCC).

### 3′RNA-seq/RNA-seq library preparation and sequencing

Total RNA for 3′RNA-seq and RNA-seq was freshly extracted using the NucleoSpin RNA isolation kit as per the manufacturer's instructions (Macherey-Nagel). The quantity and integrity of the resulting total RNA were assessed on the Agilent 2100 Bioanalyzer or TapeStation 2000 (Agilent Technologies); the RNA integrity number [RIN] was >8.0 for all samples. For RNA-seq, libraries were prepared and analyzed on the Illumina HiSeq 2000 according to the TruSeq RNA Sample Preparation Guide version 2 (Illumina) by the Molecular Genomics Core Facility (PMCC). Nine indexed samples were pooled in a single lane of a paired-end (PE50) HiSeq 2500 flow cell and sequenced on the Illumina HiSeq 2000 platform (Illumina) to generate ∼40 million paired-end 50-base-pair (bp) reads per sample. For 3′RNA-seq, libraries were prepared according to standard protocols (QuantSeq 3′ mRNA-seq FWD, Lexogen). Indexed libraries were pooled and sequenced on an Illumina NextSeq 500 (Illumina) to generate 5 million to 15 million single-end 75-bp reads per sample. For details on bioinformatics analyses, please see the Supplemental Material.

### DNA PCR assays

PCR for detection of genomic recombination across distal V_H_558 or proximal V_H_7183, V_H_Q52 regions of the IgH locus was performed using 100 ng of genomic DNA for each reaction using degenerate primers from the distal V_H_558 (5′-CGAGCTCTCCARCACAGCCTWCATGCARCTCARC-3′) or proximal V_H_7183 (VH7183 5′-CGGTACCAAGAASAMCCTGTWCCTGCAAATGASC-3′) and V_H_Q52 (VQ52 5′-CGGTACCAGACTGARCATCASCAAGGACAAYTCC-3′) regions to the J3 segment (5′-GTCTAGATTCTCACAAGAGTCCGATAGACCCTGG-3′) as described (Schlissel et al. 1991). Briefly, samples underwent denaturation for 3 min at 94°C followed by 33 cycles of 30 sec at 94°C, 30 sec at 60°C, and 1 min at 72°C and a final 10-min extension at 72°C. PCR products were separated by agarose gel electrophoresis and visualized with ethidium bromide staining.

### Statistical analysis

All analyses were performed in GraphPad Prism unless specified (GraphPad Software, Inc.), and, for all statistical tests, *P* < 0.05 was considered statistically significant. For analyses limited to two groups, the Student's *t*-test was used, and statistical significance was calculated using the Holm-Sidak method. For multiple comparisons, one-way analysis of variance (ANOVA) with Tukey's statistical hypothesis testing method was used. All Kaplan-Meier survival curves were compared using the log-rank (Mantle-Cox) test. All flow cytometry data were analyzed using FlowJo analysis software version 10.2 (Treestar).

## Supplementary Material

Supplemental Material
