# Supplementary material for: JAK2 is dispensable for maintenance of JAK2 mutant B-cell acute lymphoblastic leukemias
Source: Genes Dev. 2018 Jun 1;32(11-12):849–64. doi: 10.1101/gad.307504.117 (PMC6049517; doi:10.1101/gad.307504.117)
Supplement: Supplemental Material [file supp_32_11-12_849__index.html]

JAK2 is dispensable for maintenance of JAK2 mutant B-cell acute lymphoblastic leukemias — Supplemental Material 

# JAK2 is dispensable for maintenance of JAK2 mutant B-cell acute lymphoblastic leukemias

## Supplemental Material

- Supplemental\_Tables\_and\_Text.docx
- Supplemental\_Figures\_and\_Legends.pdf
