# Supplementary material for: JAK2 is dispensable for maintenance of JAK2 mutant B-cell acute lymphoblastic leukemias
Source: Genes Dev. 2018 Jun 1;32(11-12):849–64. doi: 10.1101/gad.307504.117 (PMC6049517; doi:10.1101/gad.307504.117)
Supplement: Supplemental Material [file supp_gad.307504.117_Supplemental_Tables_and_Text.docx]

**Supplemental Material & methods**

**Generation of Eµ-Crlf2 transgenic mice**

Eµ-Crlf2 transgenic mice were generated by pronuclear microinjection at the Water Eliza Hall Institute (Melbourne, Australia) as previously described (Brinster et al. 1981). Briefly, the cDNA sequence encoding Crlf2 was inserted downstream of the Eµ/SRα enhancer/promoter in the pEµSRα construct (Lindeman et al. 1995). The transgene was linearized and isolated from the plasmid backbone by restriction enzyme digestion (*NotI*), gel purified, microinjected into fertilized C57BL/6 CD45.2^+^ WT zygotes and transplanted into pseudo-pregnant females. Transgenic animals were identified by PCR genotyping of tail DNA using the following primers: forward (5’-CGCCGGTTGAGTCGCGTTCT-3’) and reverse (5’-GCAGGGCGCCAGTGCCATAA-3’). F1 and progenies were derived from serial backcrosses with C57BL/6 CD45.2^+^ WT mice from Eµ-Crlf2 transgenic founder D31-10.

**Murine Fetal Liver Transduction/Transplantation Model**

Fetal liver cells from embryonic day 13.5 (E13.5) fetuses of C57BL/6 CD45.1^+^ WT or Eµ-Crlf2 mice (see Supplemental Procedures for details on generation of Eµ-Crlf2 transgenic mice) were transduced two times with either MIG, MIG-Jak2^WT^, MIG-Jak2^R683G^ or MIG-Jak2^P933R^ retrovirus by spinfection as previously described (Newbold et al. 2013), in the presence of interleukin-3 (IL-3), IL-6 and stem cell factor (SCF). Prior to inoculation, 6-8 week-old male C57BL/6 CD45.1^+^ WT recipient mice were subjected to 9.0 Gy of irradiation in a split dose (4 hours apart) (Gammacell® 40 caesium source, Atomic Energy of Canada Ltd) and 0.7 × 106 transduced cells were subsequently transplanted via tail vein injection. Transplanted mice were caged in isolators and supplemented with liquid Ensure® diet (Ensure®, Abbott) for 6 weeks post-transplantation and monitored thereafter for signs of disease.

**Cell cycle and DNA content analysis**

For *in vitro* analysis, MHH-CALL4-pLMS-sh*SCR* and MHH-CALL4-pLMS-sh*JAK2*.209 cells were labeled with 10 μM BrdU *in vitro* (Thermo Fisher) for 60 minutes, washed with complete media to remove residual BrdU and fresh media was added to cells. Cells were harvested at various timepoints (0, 8, 11, 14 and 17 hr) and fixed in 2% paraformaldehyde (PFA)/PBS solution overnight (Thermo Fisher). For *in vivo* analysis, each recipient mice of Eµ-*Crlf2*/Jak2^R683G^ cells were administered with 1.0 mg BrdU/PBS solution via intra-peritoneal injection 2 hr prior to euthanizing the mice. Splenocytes were red cell lysed in ACK lysis buffer, washed in PBA solution (0.1% BSA and 0.1% NaN_3_ in PBS) and fixed overnight. Fixed cells were then incubated in 100 μL of 100 μg/mL DNAse I (Roche) in DNase buffer (1.0 mM CaCl_2_ and 1.0 mM MgSO_4_ in PBS) for 30 min at RT. Cells were washed with 1 mL PBT (0.1%BSA and 0.1% Tween20 in PBS) prior to staining with Brdu-APC antibody (Thermo Fisher; 1:50). Cells were resuspended in 1:20 7-AAD (BD Pharmingen^TM^) solution (diluted in PBT) and GFP^+^ MHH-CALL4 cells and dsRed^+^ Eµ-*Crlf2*/Jak2^R683G^ cells were analysed by flow cytometry (BD LSR Fortessa^TM^).

**RNA isolation and Quantitative Real-time PCR**

RNA was isolated using the NucleoSpin RNA isolation kit (Macherey-Nagel) as per manufacturers’ instructions. Quality and concentration of the resulting RNA was determined using a Nanodrop (ThermoScientific) and cDNA prepared using SuperScript™ reverse transcriptase and random primers (Invitrogen). Quantitative real-time PCR (qPCR) was performed by the SYBR^®^ green detection method using 100 nM each of forward and reverse primers in a 384-well plate format (Applied Biosystems). Reaction mixtures were prepared in triplicate for each cDNA sample and incubated in an Applied Biosystems 7900HT Real-Time instrument with cycle conditions consisting of 95°C for 15 minutes, followed by 40 cycles of 95°C for 30 seconds and 60°C for 30 seconds. For quantification, the C_T_ value for each sample was obtained and normalized to the C_T_ values of the *L32* gene (housekeeping gene). For specific primer sequences, please refer to supplemental table S3.

**Retroviral vector constructs and shRNA design**

The doxycycline inducible RNAi vector pREBIR (TRE3G-dsRed-miRE/shRNA-PGK-eBFP2-IRES-rtTA3) was constructed from the pREN (TRE3G-dsRed-miRE/dsRed-PGK-NeoR) vector that is based on the pQCXIX self-inactivating backbone (Fellmann et al. 2013). Using standard cloning techniques, the NeoR (neomycin resistance gene) cassette on pREN was firstly replaced with enhanced blue fluorescent protein 2 (eBFP2) from pBad-eBFP2 (Addgene plasmid 14891) creating pREB (TRE3G-dsRed-miRE/shRNA-PGK-eBFP2). IRES-rtTA3 was subsequently excised from pREVIR (Zuber et al. 2011), and cloned into pREB immediately upstream of eBFP2 creating pREBIR. Short hairpin RNAs (mir30 shRNAs) targeting murine *Jak2* and human *JAK2* were designed as previously described, and cloned into either pLMS-GFP or pREBIR-eBFP2 (Vert et al. 2006). For specific shRNA sequences see supplemental table S1.

**Intracellular staining for c-Myc**

Intracellular staining of c-Myc protein was performed as previously described (Heinzel et al. 2017). Briefly, cells were harvested at the indicated timepoints and stored in fixation buffer (0.5% paraformaldehyde, 0.2% Tween-20 and 0.1% bovine serum albumin in PBS) at 4°C for at least 16 hr until staining was performed. For staining, samples were incubated with either anti c-Myc (5605S, Cell Signalling) or a rabbit IgG isotype-matched control antibody (3900S, Cell Signalling) for 40 min at room temperature. Cells were subsequently, incubated for 40 min with an anti-rabbit IgG conjugated to Alex Fluor 568 (A11011, Thermo Fisher) and analysed by flow cytometry.

**RNA-Seq Data Analysis**

Adapter sequence, primers, poly-A tails and other short read sequences were removed from the RNA-Seq reads using Cutadapt v1.7 (Marce Martin, Science for Life Laboratory). Reads were subsequently aligned to either the human (HG19) or mouse reference genome (MM10) using the TopHat v2.0 read-mapping algorithm (Anders et al. 2015). The Voom-*limma* workflow was used for data normalisation and compute statistical significance of differential gene expression (Law et al. 2014). Multidimensional scaling (MDS) plots were generated from the Voom-*limma* workflow.

Unsupervised hierarchical clustering was performed using genes that were differentially expressed across all treatment samples according to the normalized expression matrix output from the EdgeR *limma*-Voom workflow. Samples were clustered using Pearson’s correlation (uncentered) similarity metric with average linkage on the Gene Cluster 3.0 software (de Hoon et al. 2004). Treeview 3.0 (Christopher Keil, Lewis-Sigler Institute for Integrative Genomics) was used to generate dendrograms to visualize clustered matrices. GSEA of functionally validated gene sets (obtained from the MSigDB repository) was performed using a pre-ranked gene list as previously described (Subramanian et al. 2005).

**Supplemental Table**

**Table S1: Sequence of shRNAs used in manuscript**

| Gene target (species) | Construct name | Target sequence (5’ to 3’) |
| --- | --- | --- |
| Renilla Luciferase | *Ren*.713 | CAGGAATTATAATGCTTATCTA |
| Scrambled | *SCR* | ATCTCGCTTGGGCGAGAGTAAG |
| *JAK2* (human) | **JAK2*.209 | CCAAGACAAAGAATACTATAAA |
| *JAK2* (human) | *JAK2*.2826 | AGCAGAATTAGCAAACCTTATA |
| *Jak2* (mouse) | *Jak2*.1028 | CGCAATTCAGTCAATGTAAAGC |
| *Jak2* (mouse) | *Jak2*.3323 | CGGACAAAGAATACTACAAAGT |
| *MYC* (human) | *MYC.1891* | TGTTTCAACTGTTCTCGTCGT |

*shRNA sequence was obtained from Koppikar et al. 2012

**Table S2: Sequence of sgRNAs used in manuscript**

| Gene target (species) | Construct name | Target sequence (5’ to 3’) |
| --- | --- | --- |
| *JAK2* (human) | *JAK2.9901* | AATGAAGAGTACAACCTCAG |
| *JAK2* (human) | *JAK2.9903* | CTGCCACTGCAATACCAACG |

**Table S3: Sequence of Quantitative Real-Time (qPCR) primers used in manuscript**

| Target gene | Primer sequences (5’ to 3’) |
| --- | --- |
| *Myc* | FWD: GGACGACGAGACCTTCATCAA  REV: CCAGCTTCTCTGAGACGAGCTT |
| *JAK2* | FWD: ATCTCAGATATGCAAGGGTATGG  REV: CTGTTCTCGTTCTCCACCAATA |
| *L32*  (Housekeeping gene) | FWD: TTCCTGGTCCACAACGTCAAG  REV: TGTGAGCGATCTCGGCAC |

**Supplemental References**

Anders S, Pyl PT, Huber W. 2015. HTSeq--a Python framework to work with high-throughput sequencing data. *Bioinformatics* **31**: 166-169.

Brinster RL, Chen HY, Trumbauer M, Senear AW, Warren R, Palmiter RD. 1981. Somatic expression of herpes thymidine kinase in mice following injection of a fusion gene into eggs. *Cell* **27**: 223-231.

de Hoon MJ, Imoto S, Nolan J, Miyano S. 2004. Open source clustering software. *Bioinformatics* **20**: 1453-1454.

Fellmann C, Hoffmann T, Sridhar V, Hopfgartner B, Muhar M, Roth M, Lai DY, Barbosa IA, Kwon JS, Guan Y et al. 2013. An optimized microRNA backbone for effective single-copy RNAi. *Cell Rep* **5**: 1704-1713.

Heinzel S, Binh Giang T, Kan A, Marchingo JM, Lye BK, Corcoran LM, Hodgkin PD. 2017. A Myc-dependent division timer complements a cell-death timer to regulate T cell and B cell responses. *Nat Immunol* **18**: 96-103.

Law CW, Chen Y, Shi W, Smyth GK. 2014. voom: Precision weights unlock linear model analysis tools for RNA-seq read counts. *Genome Biol* **15**: R29.

Lindeman GJ, Harris AW, Bath ML, Eisenman RN, Adams JM. 1995. Overexpressed max is not oncogenic and attenuates myc-induced lymphoproliferation and lymphomagenesis in transgenic mice. *Oncogene* **10**: 1013-1017.

Newbold A, Matthews GM, Bots M, Cluse LA, Clarke CJ, Banks KM, Cullinane C, Bolden JE, Christiansen AJ, Dickins RA et al. 2013. Molecular and biologic analysis of histone deacetylase inhibitors with diverse specificities. *Mol Cancer Ther* **12**: 2709-2721.

Subramanian A, Tamayo P, Mootha VK, Mukherjee S, Ebert BL, Gillette MA, Paulovich A, Pomeroy SL, Golub TR, Lander ES et al. 2005. Gene set enrichment analysis: a knowledge-based approach for interpreting genome-wide expression profiles. *Proc Natl Acad Sci U S A* **102**: 15545-15550.

Vert JP, Foveau N, Lajaunie C, Vandenbrouck Y. 2006. An accurate and interpretable model for siRNA efficacy prediction. *BMC Bioinformatics* **7**: 520.

Zuber J, McJunkin K, Fellmann C, Dow LE, Taylor MJ, Hannon GJ, Lowe SW. 2011. Toolkit for evaluating genes required for proliferation and survival using tetracycline-regulated RNAi. *Nat Biotechnol* **29**: 79-83.
