# Supplementary material for: JAK2 is dispensable for maintenance of JAK2 mutant B-cell acute lymphoblastic leukemias
Source: Genes Dev. 2018 Jun 1;32(11-12):849–64. doi: 10.1101/gad.307504.117 (PMC6049517; doi:10.1101/gad.307504.117)
Supplement: Supplemental Material [file supp_gad.307504.117_Supplemental_Figures_and_Legends.pdf]

**Supplementary Figure 1, related to Figure 1:**

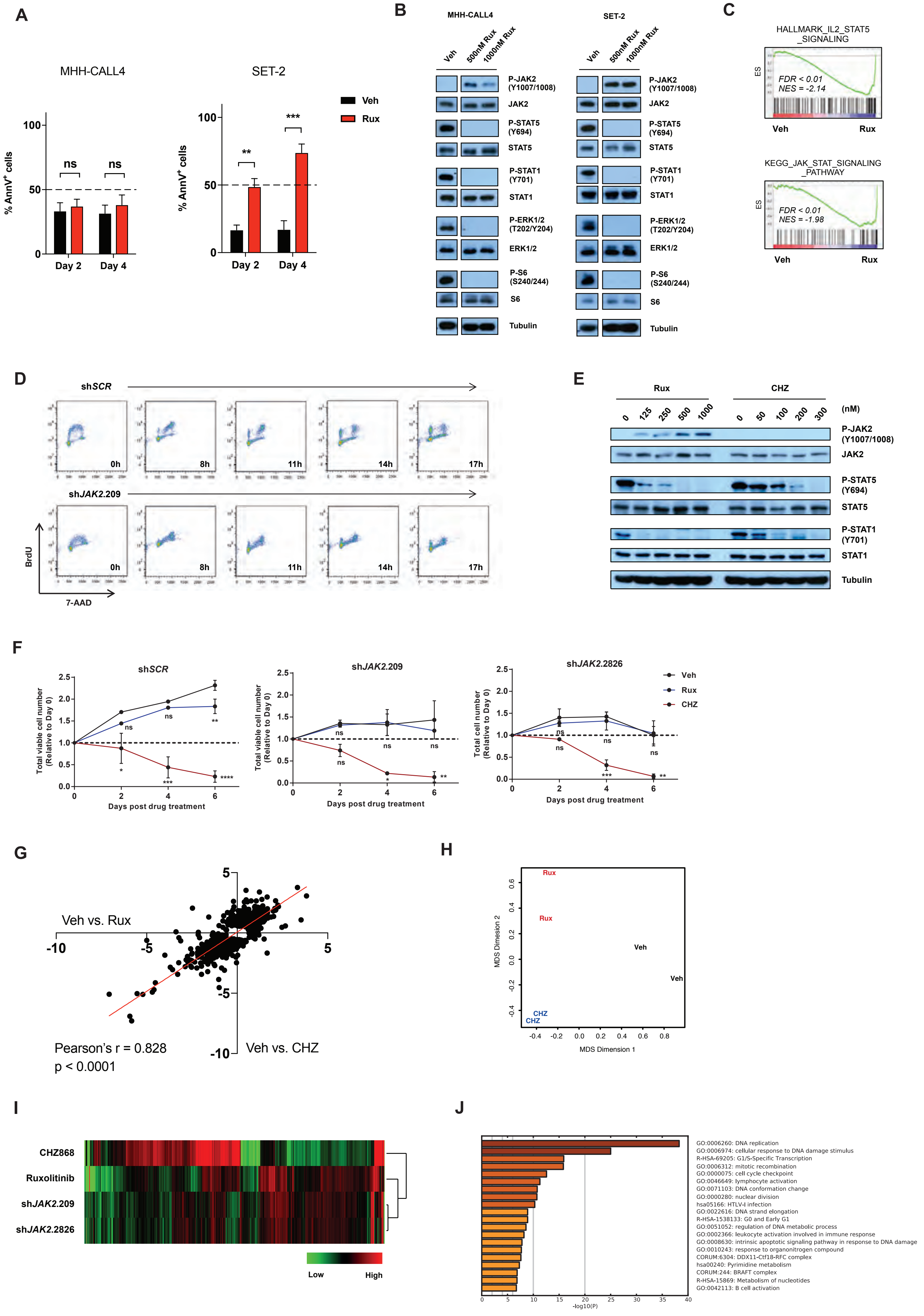

**Supplementary Figure 1, related to Figure 1.** (A) SET-2 and MHH-CALL4 cells were treated with vehicle (Veh; DMSO) or ruxolitinib (Rux; 1000 nM). Cell death was quantified by AnnV$^+$ staining at day 2 and 4-post drug treatment. Cells were passaged and drugs were replenished to the indicated concentration every 48 hr. (B) Immunoblotting against the indicated targets was performed on lysates from SET-2 and MHH-CALL4 cells treated with vehicle (DMSO) or ruxolitinib (500 or 1000 nM) for 8 hr. Tubulin served as loading control. (C) GSEA plots from 3'RNA-Seq analysis demonstrates significant negative enrichment of indicated JAK/STAT signature gene sets in MHH-CALL4 cells treated with ruxolitinib (1000 nM; 24 hr) compared to vehicle (DMSO; 24 hr). (D) Cells from Figure 1I were cultured in the absence of BrdU for the indicated time points. Flow cytometric plots (BrdU/7-AAD) show progression of cells through the cell cycle over a 17 hr time course from a representative experiment. (E) Immunoblotting against the indicated targets was performed on lysates from MHH-CALL4 cells treated for 48 hr with increasing concentrations of ruxolitinib (Rux) or CHZ868 (CHZ). Tubulin served as loading control. (F) MHH-CALL4 cells were transduced as in Figure 1B. At day 5 post-transduction, GFP-sorted cells were exposed to vehicle (Veh; DMSO), CHZ868 (CHZ; 300 nM) or ruxolitinib (Rux; 1000 nM) and relative cell counts were measured every 2 days. Drugs were replenished to the indicated concentration every 48 hr. (G-J) 3'RNA Seq analysis of MHH-CALL4 cells after 24 hr of vehicle (Veh), CHZ868 (CHZ; 300 nM) or ruxolitinib (Rux; 1000 nM) treatment. (G) Correlation between CHZ868 and ruxolitinib treatment in MHH-CALL4 cells. Genes are included for which FDR $< 0.05$, in either CHZ868 or ruxolitinib treatments; each point represents an individual gene. (H) Principle component (PC) analysis of vehicle, CHZ868 and ruxolitinib treated MHH-CALL4 cells. (I) Heatmap showing expression of all

differentially expressed genes between CHZ868 and ruxolitinib treated MHH-CALL4 cells. The same genes are displayed for MHH-CALL4-pLMS-sh*JAK2*.209 and MHH-CALL4-pLMS-sh*JAK2*.2826 relative to MHH-CALL4-pLMS-sh*SCR* cells. (J) GO-term enrichment analysis of genes displayed in (I). Fold enrichment values are represented as the minus base 10 log of their corresponding p-values. All error bars in this figure represent SEM (n=2). *$p < 0.05$, **$p < 0.01$, ***$p < 0.001$, ****$p < 0.0001$, ns: non-significant ($p > 0.05$).

Supplementary Figure 2, related to Figure 2:

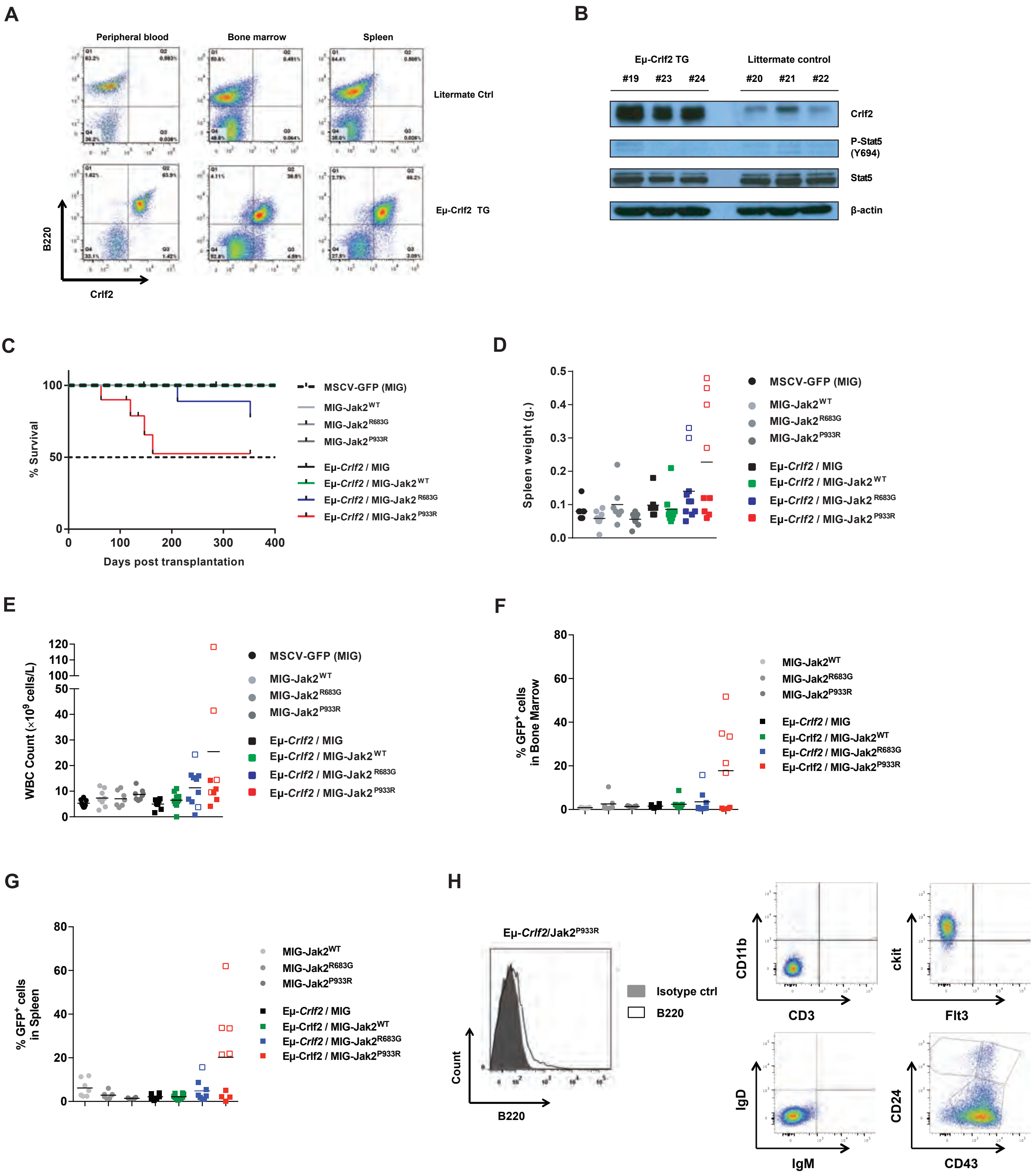

**Supplementary Figure 2, related to Figure 2.** (A) Representative flow cytometric plots showing cell surface Crlf2 expression level of B220$^{+}$ and B220$^{-}$ cells from peripheral blood, bone marrow and spleen of 6-week old Eμ-Crlf2 transgenic (#19) and littermate control mouse (#22). Endogenous Crlf2 expression level was set below the $10^3$ threshold for the purpose of this experiment. (B) 6-week old Eμ-Crlf2 transgenic (mouse ID #19, 23, 24) and littermate C57BL/6 WT CD45.2$^{+}$ control mice (mouse ID #20, 21, 22) were euthanized. Immunoblotting against the indicated targets was performed using lysates isolated from B220$^{+}$ splenocytes. β-actin served as loading control. (C) Kaplan-Meier survival curves of primary recipient mice (n=10, all groups) of Eμ-Crlf2 and C57BL/6 WT CD45.2$^{+}$ fetal liver cells transduced with MIG, MIG-Jak2$^{WT}$, MIG-Jak2$^{R683G}$ or MIG-Jak2$^{P933R}$. (D-G) Spleen weight, total WBC and the percentage of GFP$^{+}$ cells in bone marrow and spleen of individual mice from Figure S2C was analyzed at experimental endpoint. Each data point represents an individual mouse; open squares or circles denote mice that had developed leukemia. (H) Flow cytometric analysis of GFP$^{+}$ Eμ-Crlf2/Jak2$^{P933R}$ cells reveals a B220$^{low}$IgM$^{-}$IgD$^{-}$CD43$^{+}$CD24$^{-}$ckit$^{+}$CD11b$^{-}$CD3$^{-}$ immunophenotype.

**Supplementary Figure 3, related to Figure 3:**

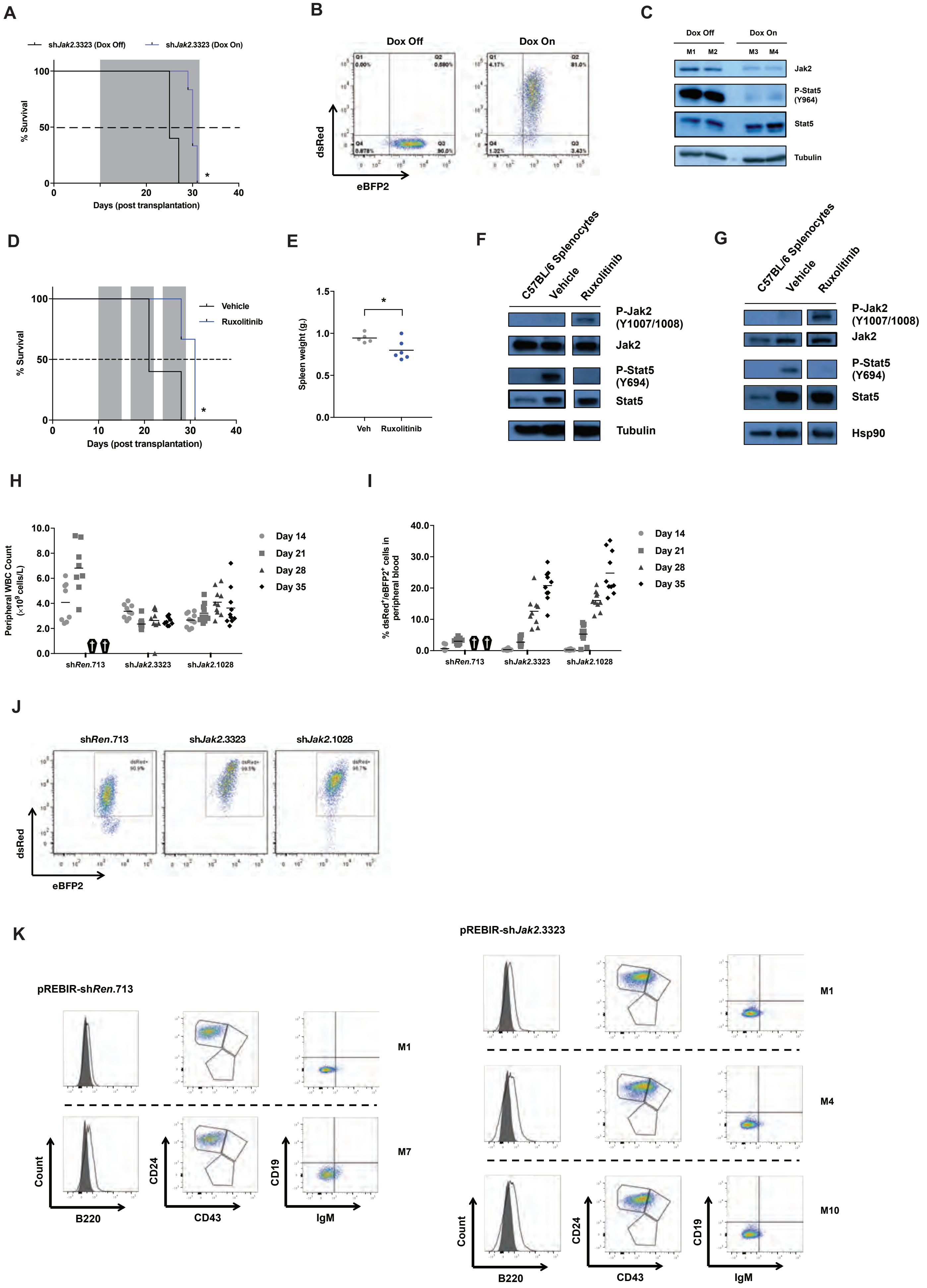

**Supplementary Figure 3, related to Figure 3**. (A) Kaplan-Meier survival curve of recipient mice of Eμ-Crlf2/Jak2$^{P933R}$-pREBIR-sh*Jak2*.3323 cells. Mice were treated with (Dox On; n=7) or without (Dox Off; n=5) doxycycline from day 10-post transplantation. Grey shading indicates period of doxycycline administration. (B) Representative flow cytometry plots showing eBFP2/dsRed proportions of GFP$^{+}$ splenocytes from moribund doxycycline-treated/untreated recipients in (A). (C) Immunoblotting for the indicated targets was performed using lysates isolated from splenocytes of doxycycline-treated (n=2; M1 & 2) / untreated (n=2; M3 & M4) recipients of Eμ-Crlf2/Jak2$^{P933R}$-pREBIR-sh*Jak2*.3323 cells at terminal disease. Tubulin served as loading control. (D) Kaplan-Meier survival curve of recipient mice of Eμ-Crlf2/Jak2$^{P933R}$ B-ALL cells treated with vehicle (0.5% methyl cellulose; n=5) or ruxolitinib (90mg/kg/2 × day; n=6). Grey shading indicates period of ruxolitinib administration. (E) At terminal disease, mice in (D) were autopsied and splenic tumour burden was assessed by weight. (F) Eμ-Crlf2/Jak2$^{P933R}$ B-ALL cells were treated *ex vivo* with vehicle (DMSO) or 1000 nM ruxolitinib for 6 hr. Immunoblotting was performed for the indicated targets using lysates isolated from treated leukemia cells and non-transformed C57BL/6 WT splenocytes. Tubulin served as loading control. (G) Eμ-Crlf2/Jak2$^{R683G}$ B-ALL cells were treated *ex vivo* with vehicle (DMSO) or 1000 nM ruxolitinib for 6 hr. Immunoblotting was performed for the indicated targets using lysates isolated from treated leukemia cells and non-transformed C57BL/6 WT splenocytes. Hsp90 served as loading control. (H-I) Total WBC analysis and flow cytometry analysis for percentage of dsRed$^{+}$/eBFP2$^{+}$ leukaemia cells (shRNA$^{+}$ cells) in peripheral blood of mice from Figure 3F was performed at the indicated timepoints. Each data point represents an individual mouse and the coffin cross sign indicates that all mice from the treatment group have

succumbed to disease at the specified timepoint. (J) Representative flow cytometry plots showing eBFP2/dsRed proportions of $GFP^+$ splenocytes from moribund doxycycline-treated recipients from Figure 3F. (K) Immunophenotyping analysis of $dsRed^+/GFP^+$ cell population from splenocytes of moribund doxycycline-treated recipient mice transplanted with Eμ-*Crlf2*/$Jak2^{R683G}$-pREBIR-sh*Ren*.713 (M#1 and M#7) and Eμ-*Crlf2*/$Jak2^{R683G}$-pREBIR-sh*Jak2*.3323 (M#1, M#4 and M#10) cells from Figure 2F at terminal disease. *$p < 0.05$.

**Supplementary Figure 4, related to Figure 5:**

**A**

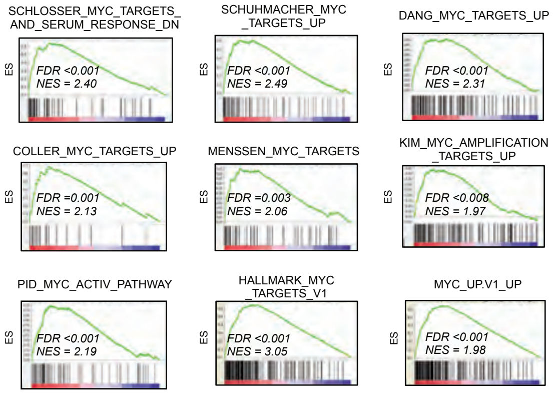


**B**

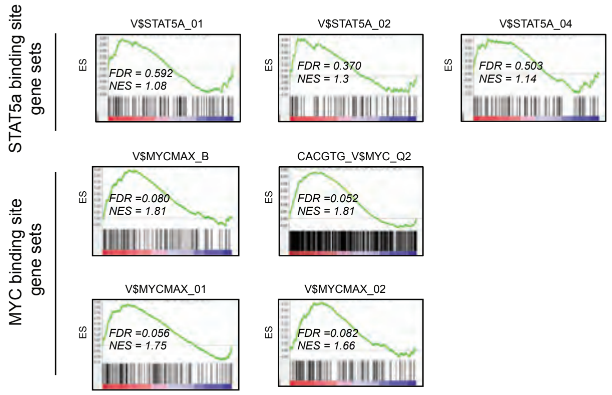


**C**

Enriched motifs in promoters of genes **induced** upon chronic relative to acute Jak2 KD

| Transcription Factor | Motif | p-value | Rank |
|---|---|---|---|
| c-Myc | 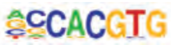 | 1e-4 | 1 |
| HEB | 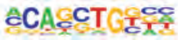 | 1e-3 | 2 |
| ZNF467 | 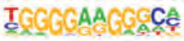 | 1e-2 | 3 |

Enriched motifs in promoters of genes **repressed** upon chronic relative to acute Jak2 KD

| Transcription Factor | Motif | p-value | Rank |
|---|---|---|---|
| KLF5 | 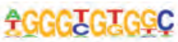 | 1e-3 | 1 |
| PU.1 | 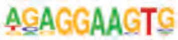 | 1e-3 | 2 |
| Sp1 | 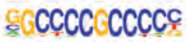 | 1e-3 | 3 |

**D**

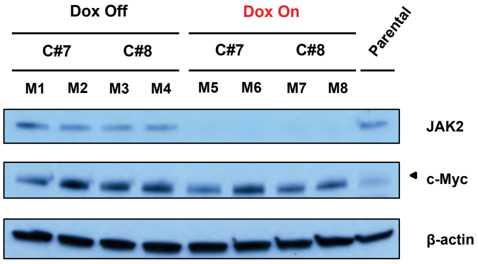

**Supplementary Figure 4, related to Figure 5**. (A-C) RNA-Seq analysis of samples generated as in Figure 5A. (A) GSEA plots of previously defined MYC gene signatures in chronic relative to acute Jak2 KD Eμ-Crlf2/Jak2$^{R683G}$ B-ALLs summarized in Figure 5D. (B) GSEA plots of previously defined gene sets with promoter regions around transcriptional start sites containing c-Myc or STAT5A motifs in chronic relative to acute Jak2 KD Eμ-Crlf2/Jak2$^{R683G}$ B-ALLs. (C) Summary of top ranked motifs enriched in promoters of genes induced or repressed upon chronic relative to acute Jak2 KD in Eμ-Crlf2/Jak2$^{R683G}$-driven B-ALL cells. The c-Myc transcription factor is denoted in red (Rank #1). (D) Immunoblotting against the indicated targets was performed using lysates isolated from parental Eμ-*Crlf2*/Jak2$^{R683G}$ cells (Parental) and those from splenocytes of individual moribund bound doxycycline-treated (Dox On; eBFP2$^{+}$/dsRed$^{+}$ cells; M5-M8) and untreated (Dox Off; eBFP2$^{+}$/dsRed$^{-}$ cells; M1-M4) recipients of Jak2 KD persistent Eμ-*Crlf2*/Jak2$^{R683G}$-pREBIR-sh*Jak2*.3323 leukemia cells (C#7 or C#8) in Figure 4B. β-actin served as loading control.

Supplementary Figure 5, related to Figure 6:

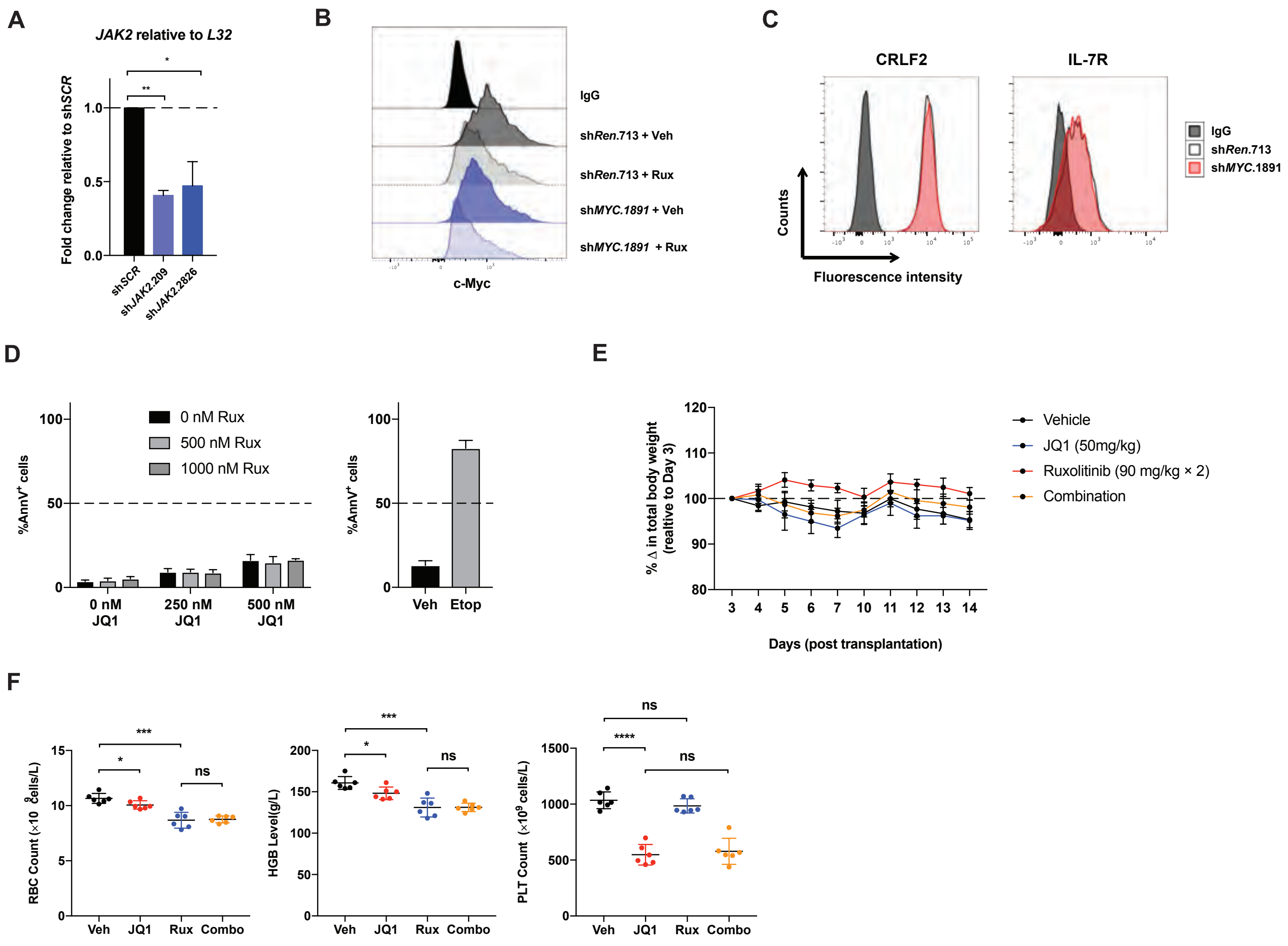

**Supplementary Figure 5, related to Figure 6**. (A) MHH-CALL4-pLMS-sh*SCR* (sh*SCR*), MHH-CALL4-pLMS-sh*JAK2*.209 (sh*JAK2*.209) and MHH-CALL4-pLMS-sh*JAK2*.2826 cells (sh*JAK2*.282) from Figure 6A were assessed for mRNA levels of *JAK2* by qPCR. Error bars represent SEM (n=2). (B) MHH-CALL4-pLMS-sh*Renilla*.713 (sh*Ren*.713) and MHH-CALL4-pLMS-sh*MYC*.1891 (sh*MYC*.1891) cells treated with vehicle (Veh; DMSO) or ruxolitinib (Rux; 1000 nM) for 24 hr, intracellular staining was performed with an antibody against c-Myc or an isotype IgG and flow cytometric analysis was performed. (C) MHH-CALL4-sh*Renilla*.713 (sh*Ren*.713) and MHH-CALL4-sh*MYC*.1891 (sh*MYC*.1891) cells were stained with an antibody against the indicated targets or an isotype IgG and flow cytometric analysis was performed. (D) MEF cells were treated with JQ1 (250 or 500 nM), ruxolitinib (Rux; 500 or 1000 nM) or combinations of both. Cell death was quantified by Annexin $V^+$ (AnnV) staining at 48 hr-post drug treatment. MEF cells were treated with etoposide (Etop; 50 μM) as positive control. Error bars represent SEM (n=3). (E) All mice from Figure 6J were weighed daily prior to drug administration (days 3-14 post transplantation. Values correspond to the average total body weight of mice ± SD in each treatment arm normalized to day 3  (n=6/group). (F) Peripheral blood analysis of haematological parameters was performed on mice from Figure 6J following 4-days of therapy. Total red blood cell count (RBC), haemoglobin level (HGB) and platelet counts (PLT) are shown. Each point represents an individual mouse. *p<0.05, **p<0.01, ***p<0.001, ns: non-significant.
